# Confidence intervals and point estimates for treatment effects in adaptive enrichment designs

**DOI:** 10.1177/09622802261423180

**Published:** 2026-02-23

**Authors:** Jinyu Zhu, Andrew Titman, Fang Wan

**Affiliations:** 1 School of Mathematical Sciences, Lancaster University, UK

**Keywords:** Clinical trials, adaptive design, subgroup analysis, interim analysis, population selection, bias

## Abstract

Adaptive enrichment designs allow subgroup selection of the patient population within a confirmatory trial via an interim analysis. However, this design complicates treatment effect estimation and uncertainty quantification. This paper introduces a p -value inversion method using various sample space orderings to construct confidence intervals either unconditionally or conditional on the subgroup selected for a general class of two-stage two-group designs. In addition, the p -value functions can be used to derive median-unbiased estimators and conditional moment estimators. Through simulation it is shown that the proposed intervals have close to nominal coverage, in contrast to naive confidence intervals based on the maximum likelihood estimator. Moreover, the median-unbiased estimators and conditional moment estimators have good performance with respect to median and mean bias, respectively. The method is illustrated by a re-analysis of a trial investigating treatment interactions with KRAS mutation type in patients with metastatic colorectal cancer.

## 1. Introduction

With the widespread adoption of human genome sequencing techniques, there is an increasing need to identify patient heterogeneity in medical practice.^1^ As a result, precision medicine has become an appealing concept in clinical treatment development and has led to the realization that the traditional one-size-fits-all approach to treatment is insufficient.^2^ Identifying the most appropriate patient population group has begun to be part of the drug development process. In order to screen out the promising population of an experimental medication, the adaptive enrichment design was introduced in Phase II/III clinical trials.^3^ The enrichment design allows for various modifications based on the interim analysis, such as sample size re-estimation and subgroup selection. However, those adaptive modifications inevitably introduce bias and difficulties in parameter inference.

There is already a large body of research on enrichment designs, such as the approach proposed by Wang et al.^4,5^ which considers adaption in sample size and futility stopping in the first interim analysis. Rather than allowing only one subgroup to be selected in the first interim analysis, the design of Magnusson and Turnbull^6^ considers cases in which more than one subgroup treatment effect exceeds the futility threshold and proceeds to subsequent stages. They assume that the sampling rule following selection is fixed. In other words, for every possible selection result, the sample size in subsequent stages should be prespecified. Based on Magnusson and Turnbull’s approach, Lin et al.^7^ proposed a design involving sample size re-estimation for stage 2 that depends on the observed statistic values in stage 1 to ensure the conditional power is maintained at a desired level. Several recent papers have considered Bayesian decision-theoretic approaches to determining the sample size and decision boundaries in enrichment designs. Ondra et al.^8^ and Burnett et al.^9^ proposed Bayesian optimal rules for subgroup selection that maximize or improve expected utilities at the interim analysis. Rosenblum et al.^10^ use sparse linear programming to optimize the decision rule for subgroup selection and multiple testing procedures.

Developing an unbiased or consistent point estimator of the treatment effect remains a significant research area because of the impact of treatment or subgroup selection characteristics in adaptive enrichment. As the naive maximum likelihood estimate fails to account for the selection bias in the initial stage, it often yields an overestimation of the actual treatment effect. Robertson et al. provide a methodological review^11^ and practical guidance^12^ on point estimation for adaptive trial designs in general. Moreover, several researchers have proposed different unbiased or bias-reduced point estimators to address the issue specifically for adaptive enrichment designs. Kimani et al.^13^ proposed two estimators for a two-stage multi-arm enrichment design, where the most effective treatment in the first stage proceeds to the second stage, and any ineffective treatments are dropped at the first stage for futility. One of the estimators is an extension of the uniformly minimum variance conditionally unbiased estimator (UMVCUE) proposed by Cohen and Sackrowitz.^14^ However, Cohen and Sackrowitz^14^ assumed that the design would always continue to the second stage, whereas Kimani et al.’s^13^ approach allows for an early stop in the first stage. The other estimator proposed by Kimani et al.^13^ is the bias-adjusted estimator, which extends the estimator proposed by Stallard and Todd.^15^ Kunzmann et al.^16^ proposed a conditional moment estimator based on the work of Luo et al.^17^ The main idea is that the conditional expectation of the statistic of the target subgroup S given interim analysis result and the observed statistic of the complimentary subgroup is a function of the true treatment effect $\theta_{S}$ and does not depend on complementary subsets. Magnusson and Turnbull^6^ evaluated the conditional and unconditional bias of the naive maximum likelihood estimate of the treatment effect and pointed out the absence of a perfectly unbiased estimator. Hence, they suggested utilizing the bootstrap method to reduce bias. Di Stefano et al.^18^ performed a simulation study to compare different methods for adjusting for selection bias in the context of adaptive enrichment designs with a time-to-event endpoint. They found that UMVCUE was most successful at removing bias, but at the cost of a high variance, resulting in the highest mean squared error (MSE), while shrinkage estimators gave the best trade-off between bias and variance to produce the lowest MSE.

The use of point estimates alone neglects the uncertainty of parameter inference, which is why many regulations mandate reporting confidence intervals for all treatment effects in clinical trials. Furthermore, the ICH E9 guideline^19^ requires that “Estimates of treatment effects should be accompanied by confidence intervals, whenever possible, and the way in which these will be calculated should be identified.” To address this, numerous studies have focussed on developing confidence interval construction for various types of adaptive designs. One such method is the confidence region approach proposed by Posch et al.^20^ for the flexible group sequential design, which utilizes the close testing procedure to adjust p -values at each stage and combines them using various combination functions. Stallard and Todd^15^ adopt the straightforward p -value inversion approach to construct confidence intervals; however, their design only allows the most effective treatment to be chosen at the interim analysis. Their p -value function is based on the ordering method proposed by Armitage^21^ and Fairbanks and Madsen^22^ which prioritizes subgroups that stop at the earlier stage for efficacy over those that stop at the later stages.

For those designs that allow flexible selection of treatment arms, Magirr et al.^23^ proposed an approach that utilizes the closed testing principle and p -value combination functions to construct a confidence region for all experimental treatment arms that strongly controls the family-wise error rate (FWER) at the desired level and is guaranteed to be concordant with the results of the hypothesis tests. Kimani et al.^24^ adopted this confidence region construction method to derive two-sided confidence intervals for time-to-event data with subgroup partition that is not prespecified but depends on the observed outcomes of patients. Nevertheless, Magirr et al.’s^23^ confidence intervals do not offer information for rejected hypotheses when just a subset of hypotheses are rejected, which potentially contributes to the conservativeness of the confidence region. Magnusson and Turnbull^6^ suggested using a double bootstrap technique for constructing confidence intervals. This approach commences with the basic maximum likelihood estimators (MLEs) and generates the initial set of bootstrap samples by simulating new datasets assuming the MLE values are correct. However, the simulation results in the paper indicated that the coverage probabilities of this method is often poor.

In this paper, we propose a p -value inversion method for the subgroup confidence interval construction similar to the approach for multiple treatment arms trial proposed by Stallard and Todd.^15^ Stallard and Todd’s^15^ method first establishes a confidence region and then reduces it to a confidence interval for the chosen treatment through two approaches: (1) assuming that the treatment effects of the unselected subgroups are equal to their MLE; (2) assuming that the treatment effects of the unselected subgroups are equal to zero. Nonetheless, the naive MLE and the null assumption overlook the bias introduced by the selection rule. Thus, we embrace a concept similar to the conditional moment estimator proposed by Luo et al.^17^ to formulate the p -value function for a subgroup by conditioning on the interim statistic for the other group(s). In enrichment designs, only subgroups with evidence of a positive treatment effect are kept following the interim analysis. Therefore, our focus lies on estimating the treatment effects for the selected group(s). Nevertheless, there is also interest in estimating the outcomes of all enrolled subgroups, but requiring adjustment for multiplicity. Hence, we construct both conditional and unconditional confidence intervals to address these considerations. In the following sections, the term “conditional” means conditioning on the event that the certain subgroup is chosen in the first stage, while the term “unconditional” refers to the process of constructing confidence intervals for the target individual subgroup regardless of the selection results in the interim analysis. In addition, our approach incorporates enrichment designs that allow more than one subgroup to be selected at the first interim analysis and the trial to be terminated early due to futility and efficacy. By inverting the p -value function derived for the confidence interval at the 0.5 significance level, we also construct the median-unbiased estimator for the enrichment design. A conditional moment estimator can also be constructed by noting that the p -value function corresponds to the conditional survivor function of the test statistic.

We focus on the class of adaptive enrichment designs that comprise two stages and two subgroups, incorporating an experimental arm and a control arm. In Section 2, we initially introduce a general form of the p -value function specific to the target subgroup, conditioning on its selection, as well as the p -value function applicable to the individual target subgroup irrespective of the selection outcome. Point estimates and confidence intervals are established using these p -value functions. The method is evaluated by simulation in Section 3. To illustrate the general method, we present a re-analysis of a clinical trial on patients with metastatic colorectal cancer in Section 4. The article concludes with a discussion.

## 2. General method of confidence interval construction and point estimate

### 2.1. Notation and setting

We assume a two-arm trial where at the first stage patients are recruited from a general patient population, but are screened to determine their membership in one of two disjoint groups j=1,2 . For instance, j=1,2 could represent biomarker positive and negative patients, respectively. More generally, a series of baseline covariates could be measured and group membership represents some known partition of the whole covariate space into two disjoint sets. The prevalence of the groups is assumed known a priori, such that if $n_{1}$ patients are planned to be recruited at the first stage then the number, $N_{1j}$ , recruited from subgroup j satisfies $E[N_{1j}|n_{1}]=\rho_{j}n_{1}$ for j=1,2 and $0<\rho_{2}=1-\rho_{1}<1$ . Patients are randomized to either the experimental treatment or the control treatment and interest lies in determining which subgroup of the patient population benefits from the new treatment. Hence, at the end of the first stage there is an interim analysis which selects a subgroup, $S^{*}$ , from S={1,2},{1},{2},∅ and determines whether to proceed to a second stage where recruitment is restricted to patients from the selected subgroup. Stopping for either futility or efficacy may also be possible.

Some designs may utilize prior knowledge of the treatment effect mechanism. For instance, if the treatment is assumed to be more promising for patients in group j=1 , then selection of $S^{*}=\{2\}$ could be precluded. Often, designs will specify a fixed stage 2 sample size assuming the trial proceeds. However, more generally, the stage 2 sample size can depend on the stage 1 data.

It is assumed that the treatment effects (experimental compared to control) for groups j=1,2 can be characterized by $\theta =(\theta_{1},\theta_{2})$ . For continuous response data, $\theta_{j}$ could represent the mean treatment difference in responses for patients in group j , for binary data, $\theta_{j}$ could represent the log-odds ratio, and for survival data $\theta_{j}$ could represent the log-hazard ratio.

Let $X_{1j}$ for j=1,2 denote the score statistic corresponding to $H_{0}:\theta_{j}=0$ . Asymptotically, $X_{1j}∼N(\theta_{j}\Delta_{1j},\Delta_{1j})$ where $\Delta_{1j}$ is the Fisher information (see for instance chapter 13.4 of Jennison and Turnbull^25^). $X_{11}$ and $X_{12}$ are assumed to be independent. In each case the alternative hypothesis to be tested is $H_{1}:\theta_{j}>0$ .

The selected subgroup, $S^{*}$ and the stage 2 Fisher information, $\left(\Delta_{21},\Delta_{22}\right)$ are assumed to be functions of $X_{1}=(X_{11},X_{12})$ . Conditional on the decision, $D=(S^{*},\Delta_{21},\Delta_{22})$ , the score statistics from the data observed in the second stage are then $X_{2j}∼N(\theta_{j}\Delta_{2j},\Delta_{2j})$ , where $X_{2j}=\Delta_{2j}=0$ if group j is not enriched at the second stage. In what follows,

$$f_{ij}(x)=\frac{1}{{\sqrt{\Delta }}_{ij}}\phi \left(\frac{x-\Delta_{ij}\theta_{j}}{\sqrt{\Delta_{ij}}}\right)$$

denotes the density of $X_{ij}$ for i,j=1,2 given $\Delta_{ij}$ .

Let $Y_{j}=X_{1j}+X_{2j}$ represent the cumulative score statistic for group j at the termination of the trial, and define the cumulative Fisher information for group j at termination as $I_{j}=\Delta_{1j}+\Delta_{2j}$ .

We can also define $X_{i0}$ to be the score statistic at stage i=1,2 corresponding to $H_{0}:\theta_{0}=0$ , where it is assumed that $\theta_{1}=\theta_{2}=\theta_{0}$ , and hence the score statistic is computed on data pooled across both groups. Asymptotically, and provided the homogeneity assumption holds, $X_{i0}∼N(\theta_{S}\Delta_{i0},\Delta_{i0})$ and, moreover, $X_{i0}$ is asymptotically equivalent to $\sum_{j\in S}X_{ij}$ , where $\Delta_{i0}=\sum_{j=1}^{2}\Delta_{ij}$ . Similarly, $Y_{0}=X_{10}+X_{20}$ is the cumulative score statistic for the whole population, with $I_{0}=I_{1}+I_{2}$ . The global statistic is also tested against a one-sided alternative, $H_{1}:\theta_{0}>0$ .

### 2.2. Framework for decisions

We assume that the adaptive enrichment design defines a mapping $d:\Omega_{0}\mapsto D$ that maps from the sample space of stage 1 score statistics, $\Omega_{0}=\{(x_{11},x_{12})\}=R^{2}$ , to a decision space consisting of $\left(S^{*},N_{2}\right)$ where $S^{*}\in \{\{1\},\{2\},\{1\}\cup \{2\}\}$ denotes the subgroup selection and $N_{2}$ is the stage 2 sample size. When $N_{2}=0$ , the trial terminates at stage 1, rejecting the null for $S^{*}$ and concluding futility for the unselected subgroup(s). It is assumed that $\left(\Delta_{11},\Delta_{12}\right)$ are known in advance.

In general, the sample space $\Omega_{0}=\{(x_{11},x_{12})\}=R^{2}$ can be partitioned into up to seven disjoint subspaces corresponding to the subspaces of D to which they are mapped:

$d(\Omega_{1})=(\emptyset ,0),$

$d(\Omega_{2})\subseteq \{D:S^{*}=\{1\},N_{2}>0\},$

$d(\Omega_{3})\subseteq \{D:S^{*}=\{2\},N_{2}>0\},$

$d(\Omega_{4})\subseteq \{D:S^{*}=\{1\}\cup \{2\},N_{2}>0\},$

$d(\Omega_{5})=(\{1\},0),$

$d(\Omega_{6})=(\{2\},0),$

$d(\Omega_{7})=(\{1\}\cup \{2\},0).$

where some designs may preclude one or more of these types of decisions leading to an empty subspace. Note that this notation differs from the used in Magnusson and Turnbull,^6^ where $\Omega_{j}$ corresponds to the set of patients in group j of the patient population.

For designs where the stage 2 sample size is not set in advance, the stage 2 information may depend on precisely where within $\Omega_{2},\Omega_{3}$ or $\Omega_{4}$ the stage 1 statistics lie, meaning that $\Delta_{21}$ and $\Delta_{22}$ are functions of $X_{1}=(X_{11},X_{12})$ .

### 2.3. Magnusson–Turnbull design

In the general case, the enrichment design proposed by Magnusson and Turnbull^6^ involves an initial stage to establish the selected subgroup, $S^{*}$ , followed by a group sequential design of an arbitrary number of stages. The design also allows for the patient population to be partitioned into an arbitrary number of subpopulations. Here we focus on the two-stage design with two subgroups.

In the first stage, the treatment effect is individually evaluated in each of the subgroups, and we only continue randomization for selected populations (i.e. subgroups with evidence of a positive treatment effect). In other words, we only use observations from the remaining subgroups when performing conditional hypothesis tests.

The choice of $S^{*}$ is based on a boundary $l_{1}$ . Specifically, group j can only be included in $S^{*}$ if $X_{1j}>l_{1}\sqrt{\Delta_{1j}}$ . Two variant decision rules are considered:

- A priori ordering: Without loss of generality, it is assumed that $\theta_{1}\geq \theta_{2}$ . In that case the trial terminates if $X_{11}\leq l_{1}\sqrt{\Delta_{11}}$ and group 2 is only included in $S^{*}$ if $X_{1j}>l_{1}\sqrt{\Delta_{1j}}$ for j=1 and j=2 . Hence the possible values of $S^{*}$ are ∅,{1} and {1,2} .
- No prior ordering: $S^{*}$ involves all groups for which $X_{1j}>l_{1}\sqrt{\Delta_{1j}}$ . Hence $S^{*}=\{1,2\}$ is also permissible.

If $S^{*}=\emptyset$ then the trial terminates. Otherwise, let $X_{1S}=\sum_{j\in S^{*}}X_{1j}$ and $\Delta_{1S}=\sum_{j\in S^{*}}\Delta_{1j}$ , then the trial stops for efficacy if $X_{1S}>u_{1}\sqrt{\Delta_{1S}}$ and proceeds to stage 2, otherwise.

At the second stage, patients will only be recruited from the selected groups. However, the total information at stage 2, $\Delta_{20}$ is assumed invariant to $S^{*}$ . The final decision at the end of stage 2 is based on the cumulative score statistic $Y_{S}=\sum_{j\in S^{*}}Y_{j}$ and corresponding cumulative Fisher information $I_{S}=\sum_{j\in S^{*}}I_{j},$ where efficacy for $S^{*}$ is declared if $Y_{S}>u_{2}I_{S}$ and the null hypothesis is accepted otherwise.

A choice can be made regarding the timing of the interim analysis, in relation to the maximum information level, $I_{max}=\sum_{i=1}^{2}\Delta_{i0}$ , for instance $\Delta_{10}=\Delta_{20}$ corresponding to equal stagewise sample sizes. The values of $l_{1},u_{1}$ and $u_{2}$ are chosen to ensure the Type I error under θ=(0,0) is equal to α , with the stage 1 boundaries set via error spending functions. The value of $I_{max}$ is then chosen to satisfy a power constraint, where the power can either be to reject the null for {1,2} or for any individual group. Full details of the calculations involved in setting the boundaries and sample size are given in Magnusson and Turnbull’s^6^ work.

**Figure 1.**
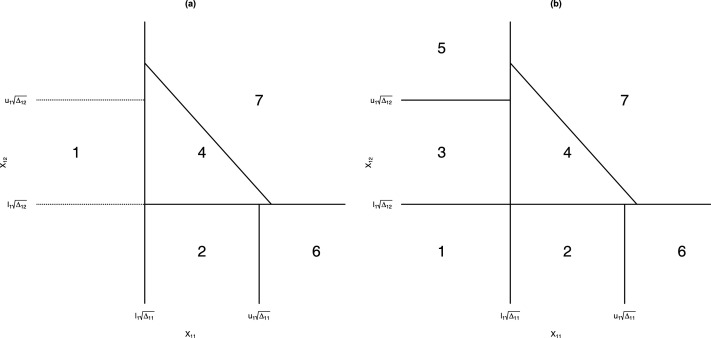
Partition of the sample space of $X_{1}$ for Magnusson and Turnbull’s design in the presence of a priori ordering (a) and without prior ordering (b).

Figure 1 illustrates the values of $X_{1}$ corresponding to $\Omega_{j}$ , j=1,…,7 , in the cases where there is a priori ordering, $\theta_{1}\geq \theta_{2}$ (left panel) and where there is no prior ordering (right panel). In the former case, the prior ordering forces $\Omega_{3}=\Omega_{5}=\emptyset$ . The stage 2 information for group j , $\Delta_{2j}$ only depends on which region $\Omega_{j}$ in which $X_{1}$ lies. Specifically

$$\Delta_{21}=\left\{ \begin{array}{ll} \Delta_{20} & \text{if}\;X_{1}\in \Omega_{2}\\ \Delta_{20}\rho_{1} & \text{if}\;X_{1}\in \Omega_{4}\\ 0 & \text{otherwise} \end{array}\right.$$

and

$$\Delta_{22}=\left\{ \begin{array}{ll} \Delta_{20} & \text{if}\;X_{1}\in \Omega_{3}\\ \Delta_{20}\rho_{2} & \text{if}\;X_{1}\in \Omega_{4}\\ 0 & \text{otherwise.} \end{array}\right.$$

In Section S2 of the Supplemental Material we show that the design of Lin *et al* (2021)^7^ also adheres to the same general framework, with the complication that the stage 2 sample size depends on the specific value of $X_{1}=(X_{11},X_{12})$ rather than just the region $\Omega_{j}$ in which $X_{1}$ lies.

### 2.4. P -value functions

Whitehead^26^ describes an approach to constructing confidence intervals based on exploiting the relationship between hypothesis testing and confidence intervals. Assuming the parameter to be estimated is denoted by θ , the general p -value function based on such relationship as p(θ,x)=Pr(X≥x;θ) where X is some summary statistic which is a random variable depending on θ , and x is the observed statistic. If the value of p(θ,x) is monotonically increasing on θ and $\theta_{\alpha }(x)$ is defined by $p(\theta_{\alpha }(x),x)=\alpha$ , then $\mathrm{Pr}(\theta \leq \theta_{\alpha }(X))=\alpha$ , which provides a method for obtaining a distinct value of θ for a given data set x with a minimum coverage probability of 1−α .

To construct a p -value function for a given parameter in the adaptive enrichment design, we consider the class of space orderings proposed by Emerson and Fleming.^27^ Specifically, using the score statistic and associated Fisher information from Section 2.1, we define a summary statistic ${\bar{Y}}_{j}=Y_{j}I_{j}^{-k},\;j=0,1,2$ and for some choice of k≥0 . Here j=0 corresponds to the case where $S^{*}=\{1,2\}.$ If k=0.5 , then ${\bar{Y}}_{j}$ is the standardized score statistic, whereas k=1 results (asymptotically) in the maximum likelihood estimate. Hence the p -value function considers the probability that ${\bar{Y}}_{j}$ would exceed the observed value ${\bar{y}}_{j}$ , considering the possibility of stopping at any stage, as a function of $\theta_{j}$ .

As noted in the introduction, interest may lie either in a confidence interval for the treatment effect in the selected subgroup $S^{*}$ or an individual component of $S^{*}$ , in which case the p -value function should consider probabilities conditional on that selection having occurred. Here, we assume that the subgroup selection occurs at the interim analysis and so a conditional confidence interval would still be computed after stage 2 even if ultimately the null hypothesis for $\theta_{S^{*}}$ was not rejected. In this way, the p -value functions do not depend on the decision boundaries of the design at the end of stage 2.

Alternatively, interest could instead lie in $\theta_{j}$ for a given group j=1,2 , regardless of whether group j was selected. In this case, simultaneous confidence intervals for the treatment effects for group 1 and 2 would be required. In what follows, we consider the two main cases, conditional or not conditional on selection, separately.

#### 2.4.1. Conditional on selection

Initially, suppose that the stage 1 data lead to a single group being chosen, such that $S^{*}=\{j\}$ for j=1 or j=2 . For the p -value function conditional on selection, ordering is with respect to ${\bar{Y}}_{j}$ and we condition on the event $S^{*}=\{j\}$ . This is equivalent to an event $X_{1}\in \Omega_{j}^{s}$ where

$$\Omega_{j}^{s}=\left\{ \begin{array}{ll} \Omega_{2}\cup \Omega_{5} & \text{if}\;S^{*}=\{1\}\\ \Omega_{3}\cup \Omega_{6} & \text{if}\;S^{*}=\{2\}. \end{array}\right.$$

The p -value function therefore concerns the probability of the event

$$\begin{array}{rl} p({\bar{y}}_{j};\theta_{j}) & =P({\bar{Y}}_{j}>{\bar{y}}_{j}|S^{*}=\{j\};\theta_{j})\\ & =P({\bar{Y}}_{j}>{\bar{y}}_{j}|X_{1}\in \Omega_{j}^{s};\theta_{j}). \end{array}$$

In the general case, $\Omega_{j}^{s}$ is not necessarily a rectangular region of $\Omega_{0}$ . As a consequence, $P(X_{j}\in \Omega_{j}^{s})$ for j=1,2 , depends on the whole vector θ . To avoid this issue, in addition to conditioning on $X_{j}\in \Omega_{j}^{s}$ , we also condition on $x_{1j^{\prime }}$ , the realized value of $X_{1j^{\prime }}$ where $j^{\prime }\neq j$ . Hence the probability of interest reduces to

$$p({\bar{y}}_{j};\theta_{j})=P({\bar{Y}}_{j}>{\bar{y}}_{j}|X_{1j}\in \Omega_{j}^{o}(x_{1j^{\prime }});\theta_{j})$$

where $\Omega_{1}^{o}(x_{12})=\{x_{1}:(x_{1},x_{12})\in \Omega_{1}^{s}\}$ and $\Omega_{2}^{o}(x_{11})=\{x_{2}:(x_{11},x_{2})\in \Omega_{2}^{s}\}$ . This is similar to the construction of the conditional moment estimator,^17^ which considers the expectation of the score statistic given the decision and the stage 1 statistic in the unselected group. Note that in the special, but common, case where $\Omega_{j}^{s}$ is a rectangular region of $R^{2}$ , $\Omega_{j}^{o}(x_{j^{\prime }})$ is invariant to the value of $x_{j^{\prime }}$ and hence the additional conditioning has no effect.

When calculating the p -value function, the stage at which the trial terminates is not conditioned upon. As a consequence, the p -value function can be written as $p=p_{1}+p_{2}$ , where the two terms correspond to the probability of exceeding the observed statistic by stopping at stage 1 for efficacy, and by proceeding to stage 2, respectively.

#### 2.4.2. Contribution of stopping at stage 1

For the contribution of stopping at stage 1, the probability of interest is

$$p_{1}({\bar{y}}_{j};S^{*}=\{j\},\theta_{j})=P[X_{1S}>{\bar{y}}_{j}\Delta_{1j}^{k}|X_{j}\in \Omega_{j}^{o}(x_{1j^{\prime }})].$$

We can first define

$$\Omega_{j}^{o1}(x_{j^{\prime }})=\left\{ \begin{array}{ll} \left\{x:(x,x_{j^{\prime }})\in \Omega_{2}\right\} & \text{if}\;j=1\\ \left\{x:(x_{j^{\prime }},x)\in \Omega_{3}\right\} & \text{if}\;j=2, \end{array}\right.$$

which represents the regions for which group j is chosen but the trial stops at stage 1, and then $\Omega_{j}^{u}(y;x_{j^{\prime }})=\Omega_{j}^{o1}(x_{j^{\prime }})\cap \{x:x>y\}$ , corresponding to the region where ${\bar{Y}}_{j}>y$ , and hence

(1) $$\begin{array}{rl} & p_{1}({\bar{y}}_{j};S^{*}=\{j\},\theta_{j})\\ & =P(X_{1j}>{\bar{y}}_{j}\Delta_{1j}^{k}|X_{1j}\in \Omega_{j}^{o1}(x_{1j^{\prime }}))\\ & =P(X_{1j}\in \Omega_{j}^{u}({\bar{y}}_{j}\Delta_{1j}^{k};x_{1j^{\prime }})|X_{1j}\in \Omega_{j}^{o1}(x_{1j^{\prime }})). \end{array}$$

Since $\Omega_{j}^{o}(x_{1j^{\prime }})$ and $\Omega_{j}^{u}(y;x_{1j^{\prime }})$ are at most a union of disjoint intervals of R and $\Omega_{j}^{u}(y;x_{1j^{\prime }})\subseteq \Omega_{j}^{o}(x_{1j^{\prime }})$ , Equation (1) can be represented by a ratio of sums of differences of normal cdfs.

#### 2.4.3. Contribution of proceeding to stage 2

For the contribution of proceeding to stage 2, let ${\tilde{x}}_{2j}(x_{1})={\bar{y}}_{j}(\Delta_{1j}+\Delta_{2j}(x_{1}){)}^{k}-x_{1j}$ represent the value of the stage 2 statistic for group j that produces the observed cumulative score statistic if $X_{1j}=x_{1j}$ and $S^{*}=\{j\}.$ The probability of interest can then be expressed as

(2) $$\begin{array}{rl} & p_{2}({\bar{y}}_{j};S^{*}=\{j\},\theta_{j},X_{1j^{\prime }}=x_{1j^{\prime }})\\ & \;=\frac{\int_{\Omega_{j}(x_{1j^{\prime }}{)}^{o2}}P[X_{2j}>{\tilde{x}}_{2j}(x_{1})|X_{1}=x_{1}]f_{1j}(x_{1j})dx_{1j}}{P(X_{1j}\in \Omega_{j}^{o}(x_{1j^{\prime }}))}, \end{array}$$

where

$$\Omega_{j}^{o2}(x_{j^{\prime }})=\left\{ \begin{array}{ll} \left\{x:(x,x_{j^{\prime }})\in \Omega_{5}\right\} & \text{if}\;j=1\\ \left\{x:(x_{j^{\prime }},x)\in \Omega_{6}\right\} & \text{if}\;j=2 \end{array}\right.$$

which represent the regions of $\Omega_{0}$ for which S is chosen but the trial proceeds to stage 2, conditional on the stage 1 statistic in the unselected group.

#### 2.4.4. P -value functions conditional on $j\in S^{*}$

For some designs, such as Lin et al’s design considered in Section S2 of Supplemental Material, the range of possible values of $x_{1j}$ given $S^{*}=j$ and given a particular $x_{1j^{\prime }}$ may not include +∞ . In those cases, rather than seeking a confidence interval for $\theta_{j}$ given $S^{*}=\{j\}$ , better-behaved confidence intervals will be obtained by conditioning only on $j\in S^{*}.$ Equally, if $S^{*}=\{1,2\}$ we could consider individual confidence intervals for $\theta_{1}$ or $\theta_{2}$ conditional on $S^{*}=\{1,2\}$ . Since in the above, we already condition on $X_{1j^{\prime }}=x_{1j^{\prime }}$ , the approach used in Section 2.4.1 can be easily adapted. It is only necessary to alter the definitions of $\Omega_{j}^{o}(x_{1j^{\prime }})$ , $\Omega_{j}^{o1}(x_{1j^{\prime }})$ and $\Omega_{j}^{o2}(x_{1j^{\prime }})$ to accommodate values that lead to either $S^{*}=\{j\}$ or $S^{*}=\{1,2\}$ . For instance, if we seek $p({\bar{y}}_{j};\theta_{j},j\in S^{*},X_{1j^{\prime }}=x_{1j^{\prime }})$ then we would take

$$\Omega_{j}^{o1}(x_{j^{\prime }})=\left\{ \begin{array}{ll} \left\{x:(x,x_{j^{\prime }})\in \Omega_{2}\cup \Omega_{7}\right\} & \text{if}\;j=1\\ \left\{x:(x_{j^{\prime }},x)\in \Omega_{3}\cup \Omega_{7}\right\} & \text{if}\;j=2, \end{array}\right.$$

whereas for $p({\bar{y}}_{j};\theta_{j},S^{*}=\{1,2\},X_{1j^{\prime }}=x_{1j^{\prime }})$ we use

$$\Omega_{j}^{o1}(x_{j^{\prime }})=\left\{ \begin{array}{ll} \left\{x:(x,x_{j^{\prime }})\in \Omega_{7}\right\} & \text{if}\;j=1\\ \left\{x:(x_{j^{\prime }},x)\in \Omega_{7}\right\} & \text{if}\;j=2. \end{array}\right.$$

#### 2.4.5. P -value functions for the common treatment effect

When $S^{*}=\{1,2\}$ , the adaptive enrichment design will typically test $H_{0}:\theta_{0}=0$ . It is therefore natural in that situation to seek a confidence interval for $\theta_{0}$ . For this purpose, we assume $\theta =(\theta_{0},\theta_{0})$ , although the consequences of making this assumption when it is not correct will be explored in Section 3.

Emulating the previous notation, define $\Omega_{0}^{s}\equiv \Omega_{4}\cup \Omega_{7}$ as the set of values of $X_{1}$ that lead to $S^{*}=\{1,2\}$ , and $\Omega_{0}^{s1}\equiv \Omega_{4}$ as the set of values for which the trial stops at stage 1 with $S^{*}=\{1,2\}$ . Then in general we can write

$$p_{1}({\bar{y}}_{0};S^{*}=\{1,2\},\theta_{0})=P(X_{1}\in \Omega_{0}^{u}({\bar{y}}_{0}\Delta_{10}^{k})|X_{1}\in \Omega_{0}^{s}),$$

where $\Omega_{0}^{u}(y)=\Omega_{0}^{o1}\cap \{(x_{1},x_{2}):x_{1}+x_{2}>y\}$ .

Similarly, let $\Omega_{0}^{o2}\equiv \Omega_{7}$ be the set of values of $x_{1}$ for which $S^{*}=\{1,2\}$ and the trial proceeds to stage 2, then

(3) $$p_{2}({\bar{y}}_{0};S^{*}=\{1,2\},\theta_{0})=\frac{\int_{\Omega_{0}^{o2}}P[X_{20}>{\tilde{x}}_{20}(x_{1})|X_{1}=x_{1}]f_{1}(x_{1})dx_{1}}{P(X_{1}\in \Omega_{0}^{s})}$$

where ${\tilde{x}}_{20}(x_{1})={\bar{y}}_{0}(\Delta_{10}+\Delta_{20}(x_{1}){)}^{k}-x_{10}$ and $f_{1}(x)=f_{11}(x_{1})f_{12}(x_{2})$ is the joint density of $X_{1}.$

Often ${\tilde{x}}_{20}(x_{1})$ and the distribution of $X_{20}$ will depend at most on $X_{10}=X_{11}+X_{12}$ , in which case (3) can be simplified to be in terms of integrals over the conditional density of $X_{10}$ given $X_{1}\in \Omega_{0}^{s}.$ This is the case in the examples considered below.

### 2.5. Unconditional p -value

Rather than considering a p -value function conditional on a given selection we may seek to construct a p -value function for $\theta_{j}$ , the treatment effect for group j=1,2 regardless of whether $j\in S^{*}$ . In order to produce a probability that only depends on the $\theta_{j}$ of interest, we again condition on $X_{1j^{\prime }}$ , the stage 1 score statistic for the other group. The ordering is with respect to ${\bar{Y}}_{j}$ , and as before the p -value function can be decomposed into two parts corresponding to group j stopping at stage 1, or group j proceeding to stage 2.

Group j could stop at stage 1 either for futility or for efficacy. Hence we first define

$$\Omega_{j}^{v}=\left\{ \begin{array}{ll} \Omega_{1}\cup \Omega_{3}\cup \Omega_{5}\cup \Omega_{6}\cup \Omega_{7} & \text{if}\;j=1\\ \Omega_{1}\cup \Omega_{2}\cup \Omega_{5}\cup \Omega_{6}\cup \Omega_{7} & \text{if}\;j=2. \end{array}\right.$$

which gives the region of $\Omega_{0}$ for which group j will stop at stage 1, and then let

$$\Omega_{j}^{v1}(y;x_{j^{\prime }})=\left\{ \begin{array}{ll} \left\{x:(x,x_{j^{\prime }})\in \Omega_{1}^{v}\}\cap \{x>y\right\} & \text{if}\;j=1\\ \left\{x:(x_{j^{\prime }},x)\in \Omega_{2}^{v}\}\cap \{x>y\right\} & \text{if}\;j=2 \end{array}\right.$$

which gives the set of values of $x_{1j}$ that lead to stopping at stage 1 with an unstandardized score statistic that exceeds y . The probability of interest is then $p_{1}({\bar{y}}_{j};\theta_{j})=P[X_{1j}\in \Omega_{j}^{v1}({\bar{y}}_{1}\Delta_{1j}^{k};x_{1j^{\prime }})].$

In order for group j to stop at stage 2, the stage 1 score statistic must lie within regions in which group j is enriched. We therefore define

$$\Omega_{j}^{v2}(x_{j^{\prime }})=\left\{ \begin{array}{ll} \left\{x:(x,x_{j^{\prime }})\in \Omega_{2}\cup \Omega_{4}\right\} & \text{if}\;j=1\\ \left\{x:(x_{j^{\prime }},x)\in \Omega_{3}\cup \Omega_{4}\right\} & \text{if}\;j=2, \end{array}\right.$$

and hence

$$p_{2}({\bar{y}}_{j};\theta_{j})=\int_{\Omega_{j}^{v2}}P[X_{2j}>{\tilde{x}}_{2j}(x_{1})|X_{1}=x_{1}]f_{1j}(x_{1j})dx_{1j},$$

where ${\tilde{x}}_{2j}(x_{1})$ is defined as in Section 2.4.1. As before, the overall p -value function is then given by $p({\bar{y}}_{j};\theta_{j})=p_{1}({\bar{y}}_{j};\theta_{j})+p_{2}({\bar{y}}_{j};\theta_{j}).$

The explicit forms of the p -value functions for the Magnusson–Turnbull design used in Sections 3 and 4 are given in the Appendix. The form of the p -value functions for Lin et al.^7^’s design is given in Section S2 of the Supplemental Material.

### 2.6. Confidence interval construction

Once the relevant p -value function has been defined for a given case, confidence interval construction then involves inverting the function. Define $A_{j}^{\alpha }({\bar{y}}_{j})=\{\theta :p({\bar{y}}_{j};\theta_{j})>\alpha \}$ for j=0,1,2 then $P(\theta_{j}\in A_{j}^{\alpha }({\bar{Y}}_{j}))=1-\alpha .$ Hence $A_{j}^{\alpha }({\bar{y}}_{j})$ serves as a 100(1−α)% confidence region for $\theta_{j}$ . Provided $p({\bar{y}}_{j};\theta_{j})$ is a monotonically increasing function in $\theta_{j}$ , there exists a unique u such that $p({\bar{y}}_{j};u)=\alpha$ and hence $A_{j}^{\alpha }({\bar{y}}_{j})=[u,\infty )$ gives a one-sided 100(1−α)% confidence interval. Moreover, if desired, $\left(u_{l},u_{u}\right)$ defined by $p({\bar{y}}_{j};u_{l})=\alpha /2$ and $p({\bar{y}}_{j};u_{u})=1-\alpha /2$ , for 0<α<0.5 , gives a two-sided 100(1−α)% confidence interval. Assuming a monotonic function, the boundaries for the confidence intervals can be computed by using a numerical line search.

For an entirely arbitrary design and an arbitrary choice of ordering parameter k , there is no guarantee that $p({\bar{y}}_{j};\theta_{j})$ increases with $\theta_{j}$ . This can occur, for instance, if score ordering is chosen ( k=0.5 ), but the stage 1 and stage 2 sample sizes are very imbalanced, and is more prone to occur for the unconditional p -values. In the context of group sequential designs, it is proven that the MLE ordering ( k=1 ) is guaranteed to lead to proper intervals whereas counter-examples exist for other orderings (Emerson and Fleming, 1990). We did not encounter any issues with the Magnusson–Turnbull design using score ordering. In contrast, implementing Lin et al’s design where the second stage sample size can be substantially larger than stage 1 led to issues using score ordering ( k=0.5 ), but was well-behaved for MLE ordering. However, if the p -value function is non-monotonic a (conservative) one-sided confidence interval could be constructed by setting the lower limit to be $\inf A_{j}^{\alpha }(\bar{y})$ . In the simulations given below, we compare these confidence to naive confidence intervals based on the MLE and Fisher information which do not account for selection. Specifically, a naive one-sided 100(1−α)% confidence interval for $\theta_{j}$ has lower bound $Y_{j}/I_{j}-\Phi^{-1}(1-\alpha )/\sqrt{I_{j}}$ .

#### 2.6.1. Simultaneous confidence intervals

Often, it will be desirable to ensure the individual confidence intervals for $\theta_{1}$ and $\theta_{2}$ collectively have 100(1−α)% coverage. Since the p -value functions for $\theta_{1}$ and $\theta_{2}$ condition on the stage 1 score statistic for the other group, $p({\bar{Y}}_{1};\theta_{1})$ and $p({\bar{Y}}_{2};\theta_{2})$ will not be independent and will have a dependence that is difficult to characterize. We therefore propose to construct simultaneous confidence intervals for $\theta_{1}$ and $\theta_{2}$ by using a Bonferroni correction. Specifically, we take $A_{1}^{\alpha /2}({\bar{y}}_{1})\times A_{2}^{\alpha /2}({\bar{y}}_{2})$ to obtain a simultaneous (1−α)100% confidence interval for $\theta =(\theta_{1},\theta_{2})$ , where we would expect the resulting confidence region to be slightly conservative. Note that this approach can be used either with the individual unconditional p -values defined in Section 2.5 or alternatively the individual p -values conditional on $S^{*}=\{1,2\}$ considered in Section 2.4.4.

### 2.7. Point estimation

While the main focus of this paper is the construction of confidence intervals for the treatment effects, the construction of the p -value function naturally also facilitates a median unbiased estimator for $\theta_{j}$ , and also gives a direct approach for calculating conditional moment estimators.

Specifically, a median unbiased estimator is given by letting ${\hat{\theta }}_{j}^{MU}$ satisfy $p({\bar{y}}_{j};{\hat{\theta }}_{j}^{MU})=0.5,$ where this approach can be applied to any of the p -value functions defined above.

Moreover, the conditional moment estimator,^16,17^ ${\hat{\theta }}_{j}^{CM}$ satisfies ${\bar{y}}_{j}=E[{\bar{Y}}_{j}|{\hat{\theta }}_{j}^{CM},S^{*},X_{1j^{\prime }}=x_{1j^{\prime }}].$ In general, we can note that $p(y;S^{*},\theta_{j},X_{1j^{\prime }}=x_{1j^{\prime }})$ is the corresponding conditional survivor distribution function of ${\bar{Y}}_{j}$ and hence

$$\begin{array}{rl} E[{\bar{Y}}_{j}|\theta_{j},S^{*},X_{1j^{\prime }}=x_{1j^{\prime }}] & =-\int_{-\infty }^{\infty }y\frac{\partial p(y;\theta_{j},S^{*},X_{1j^{\prime }}=x_{1j^{\prime }})}{\partial y}dy\\ & =\int_{0}^{\infty }p(y;\theta_{j},S^{*},X_{1j^{\prime }}=x_{1j^{\prime }})dy\\ & \;-\int_{-\infty }^{0}\left\{1-p(y;\theta_{j},S^{*},X_{1j^{\prime }}=x_{1j^{\prime }})\right\}dy. \end{array}$$

In practice, the additional integration may need to be performed numerically, making the conditional moment estimate (CME) significantly more computationally intensive to calculate than the corresponding median unbiased estimate (MUE).

An additional disadvantage of the conditional moment estimator is that in some cases it will be undefined. This can occur if the statistic in group $j^{\prime }$ is sufficiently large that given group j is chosen it is guaranteed that the procedure terminates for efficacy at stage 1. In that situation, ${\bar{Y}}_{j}$ has a lower bound at $l_{1}\Delta_{1j}^{-k}$ and $E({\bar{Y}}_{j};\theta_{j}=-\infty )>l_{1}\Delta_{1j}^{-k}$ . It is then possible to have $l_{1}\Delta_{1j}^{-k}<{\bar{Y}}_{j}<E({\bar{Y}}_{j};\theta_{j}=-\infty )$ leading to no solution for the CME equation.

In the simulations given below, we compare these point estimators with the naive maximum likelihood estimate given by ${\hat{\theta }}_{j}^{MLE}=Y_{j}/I_{j}$ where $Y_{j}$ and $I_{j}$ are the cumulative score statistic and Fisher information for $\theta_{j}$ , respectively.

## 3. Numerical studies

In this section, we evaluate the performance of confidence intervals and point estimates for Magnusson and Turnbull’s design via simulation. We consider a similar setup to the trial described in Magnusson and Turnnull’s paper,^6^ but using two rather than three subgroups. Patients in each subgroup have an equal chance of receiving either the experimental treatment or the placebo treatment. We assume patient outcomes are normally distributed with a common variance $\sigma^{2}$ , and where $\mu_{C,j}$ and $\mu_{E,j}$ denotes the expected response for subgroup j under the control and experimental treatment, respectively. Thus the true treatment effect difference in subgroup j is $\theta_{j}=\mu_{E,j}-\mu_{C,j}=\mu_{E,j}$ , and the efficient score and observed information are defined as

$$Y=({\bar{\mu }}_{E}-{\bar{\mu }}_{C})I,\text{ and }I=\frac{n}{4\sigma^{2}}$$

where ${\bar{\mu }}_{k}$ for k∈{E,C} is the sample mean of the treatment or control arm. The prevalence of subgroup 1 and subgroup 2 is 0.6 and 0.4, respectively, and we randomly generate the sample size of each subgroup by drawing from a binomial distribution. The trial is designed on the basis of a clinically relevant treatment effect of 0.2 for each subgroup, meaning a maximum of 625 patients are needed for each stage to ensure 90% power to reject the null hypothesis for at least one subgroup assuming $\theta_{1}=\theta_{2}=0.2$ , and that $\sigma^{2}=1$ , assuming a Type I error of 0.025. Utilizing the spending error functions delineated in the work of Magnusson and Turnbull,^6^ the standardized boundaries are computed as follows:

$$(l_{1},u_{1})=(0.51,2.55);(l_{2},u_{2})=(2.40,2.40).$$

Without loss of generality, $\mu_{C,j}=0$ in the simulations, meaning $\mu_{E,j}=\theta_{j}$ . We test the one-sided hypotheses $H_{0,S}:\theta_{S}=0$ and $H_{0,j}:\theta_{j}=0$ at α significance level. When evaluating the performance of confidence intervals that are conditional on a particular selected subgroup S , we use rejection sampling to obtain 10,000 trials in which $S^{*}=S.$ While for the unconditional intervals, we simply simulate 10,000 trials and retain them regardless of the selected subgroup(s). We consider seven scenarios with respect to the true treatment effects, where the first three correspond to the most anticipated outcomes - a null scenario where the target treatment causes no difference from the placebo treatment for the entire population, i.e. θ=(0,0) , a scenario where θ=(0.2,0) which means the treatment is only effective for subgroup 1 and a further scenario where (0.2,0.2) represents that the experimental treatment is effective for the entire population and treatment effect is homogeneous among them, which is also the scenario for which the design aims to have 90% power. The remaining scenarios consider less anticipated situations such as a more extreme positive treatment effect or cases where the treatment is harmful for one of the subgroups.

Let $N_{E,1j}$ and $N_{C,1j}$ be the sample size of the experimental treatment arm and the control treatment arm. All $\sigma_{0}^{2}$ s are estimated by the pooled sample variance

$${\hat{\sigma }}^{2}=\frac{\sum_{j\in \{1,2\}}(N_{E,1j}-1)S_{E,1j}+\sum_{j\in \{1,2\}}(N_{C,1j}-1)S_{C,1j}}{N_{1}-4},$$

where $S_{E,1j}$ and $S_{C,1j}$ are the sample variances of the experimental treatment arm and the control treatment arm.

### 3.1. Confidence intervals

**Figure 2.**
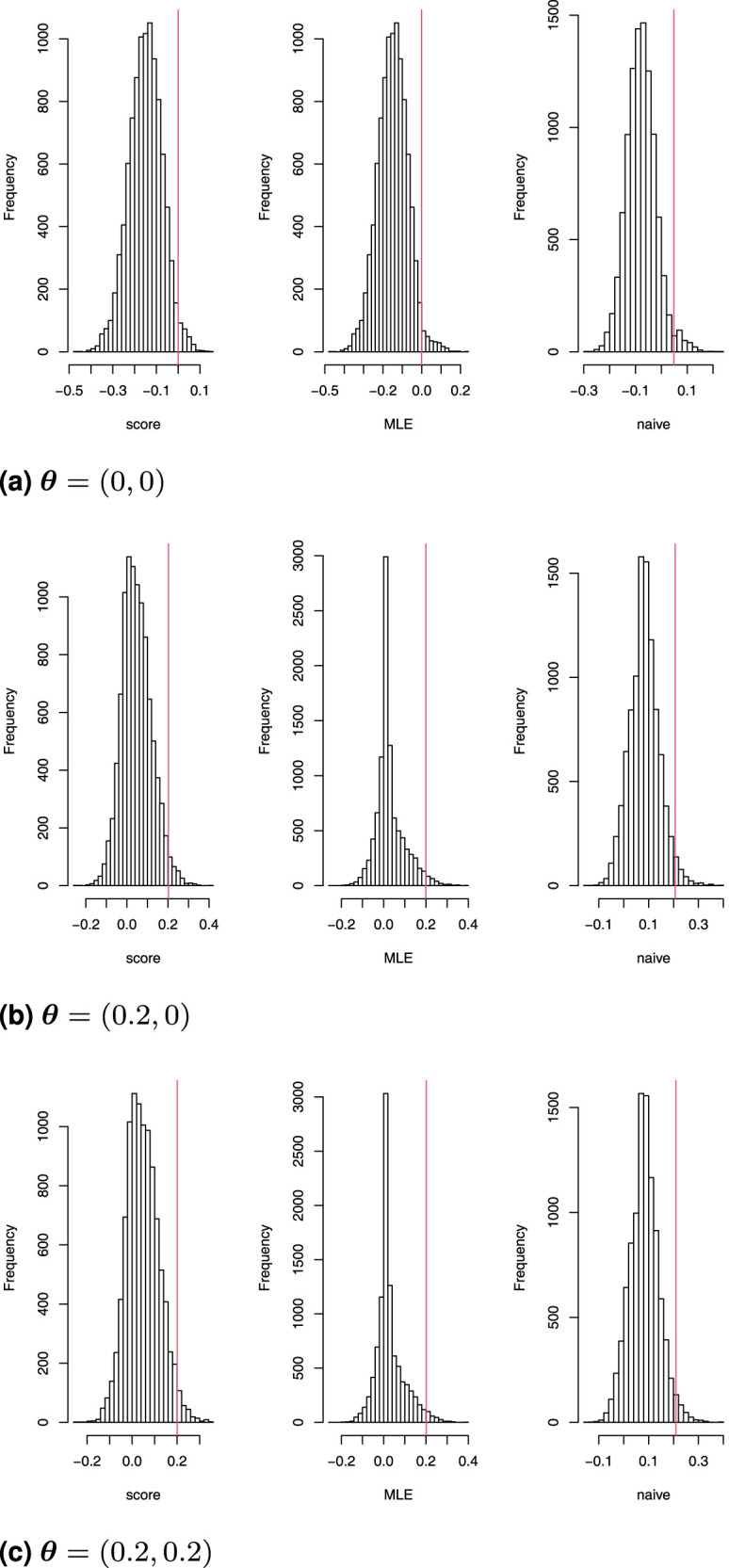
Distribution of the lower bound of a one-sided 97.5% confidence interval for $\theta_{1}$ given subgroup 1 has been selected, based on score-ordering, maximum likelihood estimator (MLE)-ordering and a naive Wald confidence interval in the Magnusson–Turnbull design. The red line is the 97.5% quantile. (a) θ=(0,0) , (b) θ=(0.2,0) and (c) θ=(0.2,0.2) .

Here, we assess the coverage properties of the proposed confidence intervals. Histograms of the distribution lower bounds under different scenarios are shown in Figure 2 given that only subgroup 1 is chosen in the first stage. Each row displays lower bounds of confidence intervals obtained under scenarios θ=(0,0) , θ=(0.2,0) and θ=(0.2,0.2) respectively. The red vertical line in each single histogram is the 97.5% quantile. Figure 2 illustrates that around 2.5% of the lower bounds, derived from both the score and MLE ordering methods, exceed the true treatment effect. This observation suggests that the coverage probability of these confidence intervals closely matches the nominal level.

Table 1 gives empirical coverage probabilities and powers of confidence intervals conditioned solely on subgroup 1 selected in the interim analysis, respectively. Here power refers to the probability that the confidence interval excludes 0 and hence coincides with Type I error for θ=(0,0) . These conditional confidence intervals are constructed using score and MLE sample space ordering approaches. In comparison to the naive confidence intervals, both the score and MLE confidence intervals exhibit coverage probabilities close to the nominal level. However, under scenarios θ=(0.2,0) and θ=(0.2,0.2) , the score ordering confidence intervals demonstrate higher power than MLE ordering confidence intervals. For the two-sided conditional confidence intervals, the results for the scenarios are also outlined in Table 1. Again, coverage probabilities demonstrate favourable performance across all scenarios. The naive confidence interval neglects any selection process during the interim analysis, leading to extremely poor coverage probability when at least one subgroup is likely to be chosen. However, its statistical power surpasses that of the conditional confidence intervals constructed through the score and MLE sample space ordering.

**Table 1.** Empirical coverage and power (type I error for null case) of conditional one-sided 97.5% and two-sided 95% CIs when subgroup 1 is selected, under different scenarios for θ in the normal distribution case.

	One-sided								
	Coverage probability			Power			Mean of lower bounds		
Scenario	Score	MLE	Naive	Score	MLE	Naive	Score	MLE	Naive
θ=(0,0)	0.9751	0.9752	0.9205	0.0249	0.0248	0.0795	−0.1496	−0.1491	−0.0771
θ=(0.2,0)	0.9732	0.9754	0.9682	0.7373	0.7218	0.9218	0.0481	0.0300	0.0829
θ=(0.2,0.2)	0.9744	0.9742	0.9683	0.7346	0.7207	0.9206	0.0482	0.0294	0.0827
θ=(0.5,0.5)	0.9751	0.9740	0.9740	0.9976	1	1	0.2419	0.2978	0.3011
θ=(0.5,0)	0.9755	0.9739	0.9739	0.9978	1	1	0.2426	0.2976	0.3011
θ=(−0.2,0)	0.9714	0.9714	0.6298	0.0008	0.0009	0.0009	−0.3538	−0.3538	−0.2162
θ=(0.2,−0.2)	0.9781	0.9741	0.9682	0.7293	0.7173	0.9171	0.0461	0.0294	0.0818
	Two-sided								
	Coverage probability			Power			Mean of CI width		
Scenario	Score	MLE	Naive	Score	MLE	Naive	Score	MLE	Naive
θ=(0,0)	0.9459	0.9494	0.9151	0.0540	0.0506	0.0848	0.2960	0.2939	0.2505
θ=(0.2,0)	0.9458	0.9447	0.9522	0.7365	0.7233	0.9145	0.4104	0.3353	0.2942
θ=(0.2,0.2)	0.9409	0.9339	0.9441	0.7275	0.7204	0.9110	0.4085	0.3349	0.2948
θ=(0.5,0.5)	0.9500	0.9495	0.9564	0.9976	1	1	0.4637	0.4056	0.4033
θ=(0.5,0)	0.9482	0.9466	0.9545	0.9978	1	1	0.4634	0.4057	0.4031
θ=(−0.2,0)	0.9433	0.9433	0.6298	0.7407	0.7408	0.2767	0.3044	0.3044	0.2479
θ=(0.2,−0.2)	0.9499	0.9459	0.9524	0.7293	0.7173	0.9171	0.4090	0.3347	0.2940

CI: confidence interval; MLE: maximum likelihood estimator.

In the scenario where both subgroups are chosen at the first interim, Table 2 reveals that the coverage probability remains close to the nominal level. However, when the treatment effect varies across subgroups, the p -value function, which assumes the treatment effects are equal, is misspecified. As a consequence, the coverage probability in relation to the population-averaged effects is somewhat below the nominal 97.5%, with this issue becoming more pronounced for the θ=(0.5,0) and θ=(0.2,−0.2) cases.

**Table 2.** Empirical coverage and power (type I error for null case) of conditional one-sided 97.5% confidence intervals when both subgroups are selected under different scenarios for θ in the normal distribution case.

	Coverage probability			Power			Mean of lower bounds		
Scenario	Score	Maximum likelihood estimator (MLE)	Naive	Score	MLE	Naive	Score	MLE	Naive
θ=(0,0)	0.9772	0.9758	0.8238	0.0228	0.0242	0.1762	−0.1495	−0.1496	−0.0429
θ=(0.2,0)	0.9681	0.9581	0.9100	0.3624	0.2683	0.8239	−0.0221	−0.0316	0.0505
θ=(0.2,0.2)	0.9719	0.9734	0.9659	0.6673	0.5172	0.9774	0.0396	0.0219	0.0939
θ=(0.5,0.5)	0.9732	0.9732	0.9732	0.9999	0.9999	1.0000	0.3394	0.3425	0.3443
θ=(0.5,0)	0.9313	0.9281	0.9259	0.9768	0.9832	1.0000	0.1747	0.1893	0.2040
θ=(−0.2,0)	0.9665	0.9665	0.4624	0.002	0.0038	0.0162	−0.2658	−0.2656	−0.1145
θ=(0.2,−0.2)	0.9134	0.8927	0.6469	0.1777	0.182	0.5365	−0.0761	−0.0750	0.0121

**Figure 3.**
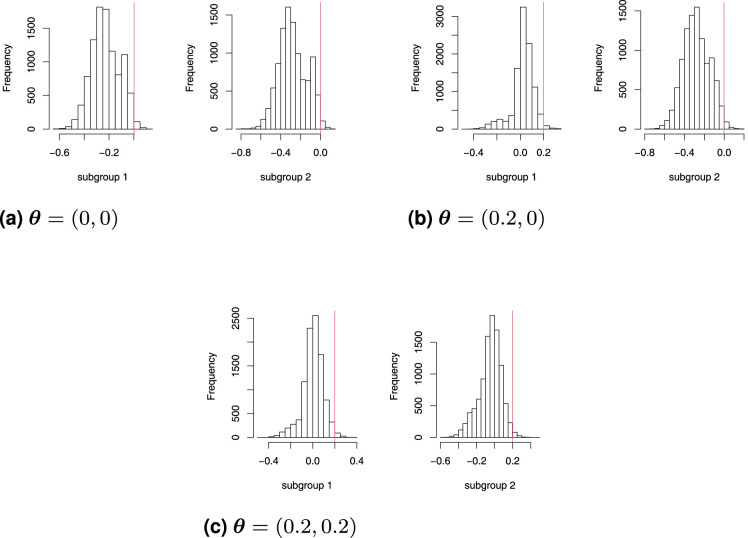
Distribution of the Bonferroni simultaneous confidence interval lower bounds with family-wise error rate (FWER) constrained at or below 0.025. The vertical red lines are the 98.75% quantiles. (a) θ=(0,0) , (b) θ=(0.2,0) and (c) θ=(0.2,0.2) .

The simultaneous confidence intervals for both subgroups are constructed using the Bonferroni approach outlined in Section 2.6 where the significance level assigned to each subgroup is α/2=0.0125 . Table 3 compares the FWER, overall power, and average number of rejections in each trial of three scenarios. We notice that all of those FWERs are close to the nominal level we desired, but not all of them are smaller than 0.025. Theoretically, by adopting the classic Bonferroni correction, the FWER should be slightly conservative. However, under the null scenario, the coverage of the 97.5% confidence is slightly below the nominal level. This is likely to be due to the intervals not accounting for the random variation in the observed subgroup prevalence or that the pooled sample variance is used in the statistic rather than the true population value of $\sigma^{2}$ . Moreover, in a single trial, score ordering simultaneous confidence intervals reject more hypotheses compared to MLE-ordered simultaneous confidence intervals, consistent with its superior overall power performance. Histograms for the distribution of the simultaneous confidence interval lower bounds are presented in Figure 3. The left histogram lists all lower bounds from subgroup 1 simultaneous confidence intervals and the right histogram lists those from subgroup 2. What can be seen in Figure 3 is that the 98.75% quantiles (vertical red line) are approximately located around the true treatment effect for every case which also implies that our individual p -value functions ensure the individual confidence intervals have coverage probabilities close to the nominal level. As for the conditional simultaneous confidence intervals, Table 4 tells that the coverage probabilities are still close to the nominal level we desire under both score and MLE orderings, but the score ordering confidence intervals have greater power.

**Table 3.** Coverage and power of unconditional simultaneous confidence intervals for $\theta =(\theta_{1},\theta_{2})$ .

	Coverage probability		Power		Average rejection		Mean of lower bounds			
							Subgroup 1		Subgroup 2	
Scenario	Score	Maximum likelihood estimator (MLE)	Score	MLE	Score	MLE	Score	MLE	Score	MLE
θ=(0,0)	0.9737	0.9737	0.0263	0.0263	0.0263	0.0264	−0.2270	−0.2283	−0.2778	−0.2798
θ=(0.2,0)	0.9752	0.9734	0.7235	0.7150	0.7289	0.7194	0.0250	0.0142	−0.2787	−0.2795
θ=(0.2,0.2)	0.9758	0.9773	0.7741	0.7549	0.9246	0.8627	0.0043	0.0019	−0.0476	−0.0535
θ=(0.5,0.5)	0.9746	0.9746	0.9997	0.9999	1.9519	0.0264	0.2691	0.2694	0.2170	0.2170
θ=(0.5,0)	0.9750	0.9757	0.9999	0.9999	1.0112	0.7193	0.2321	0.2717	−0.2831	−0.2831
θ=(−0.2,0)	0.9778	0.9777	0.0120	0.0120	0.0120	0.0120	−0.4325	−0.4321	−0.2776	−0.2809
θ=(0.2,−0.2)	0.9747	0.9741	0.7746	0.7747	0.7746	0.7747	0.0319	0.0195	−0.4840	−0.4835

Power refers to the proportion of intervals that exclude 0 for at least one component. Average rejection refers to the mean rejections of the null hypothesis in every trial.

**Table 4.** Coverage and power of conditional simultaneous confidence intervals for $\theta =(\theta_{1},\theta_{2})$ when $S^{*}=\{1,2\}$ .

	Coverage probability		Power		Mean of lower bounds			
					Subgroup 1		Subgroup 2	
Scenario	Score	Maximum likelihood estimator (MLE)	Score	MLE	Score	MLE	Score	MLE
θ=(0,0)	0.9742	0.9775	0.0258	0.0225	−0.2302	−0.2322	−0.2905	−0.2929
θ=(0.2,0)	0.9703	0.9756	0.4355	0.2922	−0.0206	−0.0420	−0.4553	−0.4634
θ=(0.2,0.2)	0.9734	0.9768	0.5091	0.3906	−0.0673	−0.0902	−0.1888	−0.2130
θ=(0.5,0.5)	0.9758	0.9758	0.9980	0.9986	0.2629	0.2631	0.1954	0.1955
θ=(0.5,0)	0.9753	0.9753	0.9865	0.9921	0.2552	0.2657	−0.7173	−0.7178
θ=(−0.2,0)	0.9726	0.9799	0.0129	0.0057	−0.4320	−0.4320	−0.2651	−0.2658
θ=(0.2,−0.2)	0.9705	0.9736	0.4579	0.2759	−0.0082	−0.0285	−0.6359	−0.6362

Power refers to proportion of intervals which exclude 0 for at least one component.

### 3.2. Point estimates

In this section, we present the outcomes of the MUE for the treatment effect, obtained by inversely applying the associated p -value functions at the 0.5 significance level and also the CME obtained by treating the p -value function as the conditional survival distribution of the test statistic. These estimates are compared to the naive maximum likelihood estimate (MLE). Tables 5 and 6 present the mean and median bias and root-mean square error (RMSE) of point estimators of the treatment effect when just subgroup 1 and when both groups are selected. In all circumstances, the median bias of the MUE is close to zero and is generally nearer than either the corresponding CMEs or naïve maximum likelihood estimates (MLEs). However, CMEs perform best in terms of mean bias. The naïve MLE usually overestimates the treatment effect as its bias is mostly positive.

**Table 5.** Performance of point estimators for $\theta_{1}$ when subgroup 1 is selected.

	Mean bias			Median bias			RMSE		
Scenario	MUE	CME	MLE	MUE	CME	MLE	MUE	CME	MLE
θ=(0,0)	−0.0002	−0.0017	0.0479	−0.0006	−0.0028	0.0040	0.0781	0.0782	0.0794
θ=(0.2,0)	0.0237	0.0184	0.0295	−0.0004	−0.0031	0.0191	0.1094	0.1027	0.0884
θ=(0.2,0.2)	0.0227	0.0178	0.0299	−0.0050	−0.0070	0.0177	0.1103	0.1039	0.0909
θ=(0.5,0.5)	−0.0015	−0.0054	0.0002	−0.0015	−0.0035	−0.0014	0.1025	0.1063	0.0997
θ=(0.5,0)	0.0016	−0.0023	0.0031	−0.0004	−0.0022	−0.0003	0.1022	0.1059	0.0997
θ=(−0.2,0)	−0.0020	−0.0027	0.1068	−0.0029	−0.0034	0.1060	0.0780	0.0775	0.1188
θ=(0.2,−0.2)	0.0107	0.0012	0.0295	0.0003	−0.0100	0.0189	0.0857	0.0861	0.0868

MUE and CME are computed based on p -value functions using MLE ordering ( k = 1). MUE: median unbiased estimate; CME: conditional moment estimate; MLE: naive maximum likelihood estimate; RMSE: root mean square error.

**Table 6.** Performance of point estimators for $\theta_{S}$ when both subgroups are selected.

	Mean bias			Median bias			RMSE		
Scenario	MUE	CME	MLE	MUE	CME	MLE	MUE	CME	MLE
θ=(0,0)	−0.0002	−0.0007	0.0479	0.0021	−0.0016	0.0656	0.0781	0.0782	0.0793
θ=(0.2,0)	0.0205	0.0192	0.0585	0.0138	0.0183	0.0496	0.0857	0.0824	0.0910
θ=(0.2,0.2)	0.0089	0.0051	0.0318	0.0003	−0.0086	0.0291	0.0829	0.0789	0.0697
θ=(0.5,0.5)	0.0006	0.0001	0.0008	0.0010	0.0010	0.0011	0.0805	0.0812	0.0800
θ=(0.5,0)	0.0551	0.0514	0.0590	0.0554	0.0524	0.0566	0.0908	0.0907	0.0894
θ=(−0.2,0)	0.0055	0.0021	0.1177	0.0048	0.0022	0.1160	0.0807	0.0785	0.1275
θ=(0.2,−0.2)	0.0480	0.0397	0.0953	0.0323	0.0189	0.0755	0.1115	0.1092	0.1241

MUE and CME are computed based on p -value functions using MLE ordering (k = 1). Assumed true value of $\theta_{S}=0.12$ used when θ=(0.2,0) , $\theta_{S}=0.30$ when θ=(0.5,0) , $\theta_{S}=-0.12$ when θ=(−0.2,0) and $\theta_{S}=0.04$ when θ=(0.2,−0.2) . MUE: median unbiased estimate; CME: conditional moment estimate; MLE: naive maximum likelihood estimate; RMSE: root mean square error.

However, while reducing bias, there is often a trade-off with the performance of RMSE. We notice that there are cases where both bias and RMSE are big, such as the conditional MLE under the null scenario. This is due to the significant bias present in this scenario (i.e. RMSE is the sum of the variance and squared bias). Additionally, when there is heterogeneity in treatment effects, the estimate of the treatment effect exhibits the highest bias and RMSE among all three estimators. This is also a consequence of the homogeneity assumption we employ in the p -value function.

A similar set of simulations based upon the design of Lin et al (2021) is presented in Section S2.2 of the Supplemental Materials.

## 4. Illustrative Example: Panitumumab-FOLFIRI versus FOLFIRI alone in patients with metastatic colorectal cancer

As a realistic motivating example, we re-analyse data from a randomized phase 3 trial on the use of FOLFIRI with panitumumab compared to FOLFIRI alone as a second-line treatment of metastatic colorectal cancer.^28,29^

The original trial (20050181) was initially designed as a conventional parallel group design, unselected by KRAS mutation status. However, emerging KRAS data from other studies of panitumumab indicated that monotherapy clinical benefit was isolated to patients with wild-type KRAS. As a consequence, the protocol was amended after completion of enrolment to incorporate patient stratification by KRAS status. Were information on the impact of KRAS status and recent advancements in adaptive enrichment design methods known at the onset of the trial, it may have been more appropriate to design the trial as a two-stage adaptive enrichment design. Given there is an a priori assumption of higher efficacy among those with wild-type KRAS, it would make sense to only continue to the second stage if there is evidence of a survival benefit for wild-type KRAS patients using panitumumab + FOLFIRI, but select the whole population if there is also evidence of a promising treatment effect for those without wild-type mutations.

Following the assumptions made in the original protocol amendment, we assume that 55% of patients are of wild-type KRAS tumour time and that a hazard ratio of 0.67 with respect to the primary endpoint of progression-free survival represents a clinically relevant treatment difference. Using a two-stage Magnusson–Turnbull design, aiming for a 90% power to reject the null hypothesis for either wild-type KRAS tumours or the whole population, assuming the clinically relevant effect holds for the whole population, controlling Type I error at 1% and assuming equal information weights before and after the interim, leads to decision boundaries $\left(l_{1},u_{1},u_{2})=(0.519,2.748,2.616\right)$ , where the maximum cumulative Fisher information requirement is 102.3.

Since patients are randomized equally to treatment groups, the Fisher information after r events have been observed is approximately r/4 .^30^ Hence the interim analysis should occur after 205 events have occurred (from either KRAS tumour type). Using the potential follow-up time variable in the dataset to infer relative recruitment times, the interim analysis would occur 382 days after the first patient was randomized. At this point the respective log-rank Z -statistics are 2.73 for the wild type and -0.17 for the non-wild type. Hence, based on the Magnusson–Turnbull design, while there is strong evidence of a treatment effect in the wild-type subgroup it is just below the stopping threshold, $u_{1}=2.748$ . Hence the trial would proceed to a second stage where subsequent patients would only be enrolled if their tumour is of wild-type and the final analysis occurs after a further 205 events (among wild-type tumour patients recruited at either stage). Taking these patients from the remaining wild-type tumour patients in the original trial, the final analysis would occur at 664 days, where the final Z-statistic is 2.670. Hence the conclusion is that there is survival benefit of the combination treatment for wild type tumours (since $2.67>u_{2}=2.616$ ). The stagewise results of the trial are given in Table 7.

**Table 7.** Results of the panitumumab-FOLFIRI trial run as a two-stage Magnusson and Turnbull design.

Stage 1	$X_{1,j}$	$R_{1,j}$	$\Delta_{1,j}$	${\bar{X}}_{1,j}$
Wild type	13.04	94	22.80	2.73
Not wild type	-0.87	111	26.29	-0.17
Stage 2	$X_{2,1}$	$R_{2,1}$	$\Delta_{2,1}$	
Wild type	9.94	207	51.26	
Total	$Y_{2,1}$	$I_{2,1}$	$Y_{1,j}/\sqrt{I_{2,1}}$	$Y_{1,j}/I_{2,1}$
Wild type	22.98	74.06	2.67	0.31

$R_{i,j}$ refers to the number of events in group j at stage i .

In order to implement the methods in Section 2, we make the approximation (which holds asymptotically) that the score (log-rank) statistic is $X_{ij}∼N(\theta_{j}\Delta_{ij},\Delta_{ij})$ such that $Y_{ij}/I_{ij}$ can be used as an estimator for $\theta_{j}$ and also approximates the Cox partial likelihood MLE.

When a subgroup stops before stage 2, the corresponding p -value function requires an estimate of the stagewise information which would have been observed had the trial proceeded (and conditional on the stage 1 result for the other subgroup). For normally distributed response data and assuming the stage two sample size were adhered to, it is reasonably uncontroversial to use the estimate of the pooled residual variance at stage 1 to estimate the counterfactual stage two information. For survival data the correct way to estimate the stage 2 information is less clear. Here, we take the convention that the rate of stage 2 information per observed event is the same as observed in stage 1. For instance, if the same number of events are to be observed in each stage, the stage 2 information should be equal to that of stage 1. Therefore, if subgroup j is chosen on its own but stops for efficacy at stage 1, the potential stage 2 information for group j (had the trial proceeded to stage 2) is taken as $\Delta_{11}+\Delta_{12}$ . Similarly, if both groups are chosen and the trial stops at stage 1, the stage 2 information for group j is taken as $\Delta_{1j}$ .

The 95% confidence interval for the log-hazard ratio of wild type KRAS tumour patients, conditional on selection, using MLE ordering ( k=1 ) is ( − 0.526, − 0.015), corresponding to a HR of between 0.59 and 0.99. The median unbiased estimator is − 0.284, while the conditional moment estimator is − 0.260. These contrast to the uncorrected Cox proportional hazards model MLE which is − 0.309 (95% CI: − 0.536, − 0.082), which is itself very close to the approximate uncorrected estimate $-Y_{1,j}/I_{2,1}=-0.31$ .

The simultaneous unconditional 95% confidence intervals for the log-hazard ratios for wild type and non-wild type tumours are (−0.609,−0.036) and (−0.404,0.461) , respectively, which in this case, broadly agrees with the conclusions of the trial. To compute the unconditional p -value function for non-wild type tumours the counterfactual stage 2 information is taken to be equal to that group’s stage 1 information.

In Section S1 of the Supplemental Materials, additional simulations investigate the Magnusson–Turnbull designs for a time-to-event endpoint, where it is shown that performance comparable to the normally distributed case can be achieved for the confidence intervals and point estimators.

## 5. Discussion

In this paper, we have shown that confidence intervals, both conditional and unconditional on subgroup selection, can be constructed for adaptive enrichment designs by use of p -value function inversion. Unlike naive confidence intervals based on the MLE and Fisher information, our proposed intervals have close to nominal coverage in most cases. The exception is when $\theta_{1}\neq \theta_{2}$ but $S^{*}=\{1,2\}$ . In that case, it was assumed that $\theta_{1}=\theta_{2}$ in order to obtain a confidence interval for the overall population effect but the simulations indicated that when $\theta_{1}\neq \theta_{2}$ , the confidence interval for $\theta_{0}$ assuming homogeneity will have less than nominal coverage for the population effect $\theta_{0}=\rho_{1}\theta_{1}+(1-\rho_{2})\theta_{2}$ , and it is a remaining open problem how to construct a confidence interval for $\theta_{0}$ in that situation. Nevertheless, when $S^{*}=\{1,2\}$ it is also possible to construct simultaneous confidence intervals for $\theta_{1}$ and $\theta_{2}$ which were shown to have close to nominal simultaneous coverage even when $\theta_{1}\neq \theta_{2}$ .

The constructed p -value functions were also shown to provide both a MUE and CME. Through simulation, these estimators were shown to be effective at providing estimates with low median-bias, or mean-bias, for MUE and CME, respectively. Nevertheless, in many cases, the naive MLE may be comparable or superior on the basis of RMSE.

Throughout the paper, a trial with two stages and two subgroups is assumed. Assuming, the subgroup selection still occurs at the end of the first stage, the methods can be extended to either designs with more than two subgroups or trials with more than two stages, assuming subgroup selection occurs at the end of the first stage. If there are J>2 groups then the sample space of $X_{1}$ will be in J dimensions and the possible decision space will involve partitioning into a higher number of regions. As in the two-stage case, p -value functions can be computed by considering, $p=\sum_{i=1}^{I}p_{i}$ , where $p_{i}$ is the probability of exceeding ${\bar{y}}_{j}$ and stopping at stage i , for i=1,…,I . However, in general, the calculation of $p_{i}$ requires an increasing dimension of integration as i increases.

A limitation of the proposed confidence intervals is that they rely on asymptotic approximations for the distribution of the score statistic. Generally, these approximations will perform well for continuous endpoints with moderate sample sizes. Potentially, the methods in this paper could also be extended to assume a non-central t -statistic for the score statistic to allow robustness to even lower sample sizes. However, for time-to-event data, the expected Fisher information depends on the treatment effects θ whereas our method assumes the Fisher information is fixed. Potentially, a larger sample is therefore needed to achieve accuracy. However, in Section S1 of the Supplemental Materials the intervals are shown to perform well for a realistically sized trial powered to obtain 80% power to detect a hazard ratio of 0.74 (log HR = -0.3).

Ideally, confidence intervals in adaptive enrichment trials would have concordance with the trial conclusion. For trial designs involving a closed testing procedure and using a p -value combination formulation to combine data across the two stages, it should be possible to adapt the approach of Magirr et al.^23^ to produce concordant simultaneous intervals, although it is unclear whether they would lead to informative intervals. Our method aims to be general and to provide informative intervals but has the limitation of having no guarantee of concordance. Potentially, the degree of disagreement could be reduced by judicious choice of the ordering parameter k . For instance, in Magnusson and Turnbull’s design score-ordering ( k=0.5 ) leads to disagreement due to the design thresholds, $u_{1}$ and $u_{2}$ being different. Choosing k such that $u_{1}I_{1,j}^{0.5-k}=u_{2}I_{2,j}^{0.5-k}$ removes this form of disagreement, except that the value of k would depend on the group j under consideration.

Functions in **R** to obtain confidence intervals as well as CME and MUEs for both the Magnusson–Turnbull design and the Lin *et al* design are provided in the Supplemental Materials. Our method can be applied to nearly all adaptive enrichment designs that specify subgroups in advance. However, further research is needed to develop a more comprehensive approach capable of accommodating designs like the one proposed by Simon and Simon,^31^ where subgroups are not predetermined.

## Supplemental Material

sj-pdf-1-smm-10.1177_09622802261423180 - Supplemental material for Confidence intervals and point estimates for treatment effects in adaptive enrichment designs Supplemental material, sj-pdf-1-smm-10.1177_09622802261423180 for Confidence intervals and point estimates for treatment effects in adaptive enrichment designs by Jinyu Zhu, Andrew Titman and Fang Wan in Statistical Methods in Medical Research

sj-pdf-2-smm-10.1177_09622802261423180 - Supplemental material for Confidence intervals and point estimates for treatment effects in adaptive enrichment designs Supplemental material, sj-pdf-2-smm-10.1177_09622802261423180 for Confidence intervals and point estimates for treatment effects in adaptive enrichment designs by Jinyu Zhu, Andrew Titman and Fang Wan in Statistical Methods in Medical Research

## Appendix: Point estimates and confidence intervals for the Magnusson–Turnbull design

In this section the methods considered in Section 2.4 are applied directly to the Magnusson–Turnbull design introduced in Section 2.3. We specifically present the variant of the design where no prior ordering is assumed. However, the results are easily adapted to the case of a prior ordering.

*Conditional p -values for $\theta_{1}$
*

Suppose firstly that $S^{*}=\{1\}$ , then $\Omega_{1}^{o}=(l_{1}\sqrt{\Delta_{11}},\infty )$ and $\Omega_{1}^{u}({\bar{y}}_{1}\Delta_{11}^{k};x_{12})=(u_{1}\sqrt{\Delta_{11}}\vee {\bar{y}}_{1}\Delta_{11}^{k},\infty ),$ and hence

$$\begin{array}{rl} p_{1}({\bar{y}}_{1};S^{*}=\{1\},\theta_{1})= & P(X_{11}>(u_{1}\sqrt{\Delta_{11}}\vee {\bar{y}}_{1}\Delta_{11}^{k})|X_{11}>l_{1}\sqrt{\Delta_{11}})\\ = & \frac{1-\Phi ((u_{1}\vee {\bar{y}}_{1}\Delta_{11}^{k-0.5})-\theta_{1}\sqrt{\Delta_{11}})}{1-\Phi (l_{1}-\theta_{1}\sqrt{\Delta_{11}})}, \end{array}$$

where there is no direct dependence on the specific value of $X_{12}$ . Similarly, $\Omega_{1}^{o2}=\{x_{1}:l_{1}\sqrt{\Delta_{11}}<x_{1}\leq u_{1}\sqrt{\Delta_{11}}\}$ and if $S^{*}=\{1\}$ then $\Delta_{21}=\Delta_{20}$ . Hence,

(A.1)
$$\begin{array}{rl} p_{2}({\bar{y}}_{1};S^{*}=\{1\},\theta_{1}) & =\frac{\int_{l_{1}\sqrt{\Delta_{11}}}^{u_{1}\sqrt{\Delta_{11}}}P(X_{21}>{\bar{y}}_{1}(\Delta_{11}+\Delta_{2}{)}^{k}-x_{11})f_{11}(x_{11})dx_{11}}{1-\Phi (l_{1}-\theta_{1}\sqrt{\Delta_{11}})}\\ & =\frac{\int_{l_{1}\sqrt{\Delta_{11}}}^{u_{1}\sqrt{\Delta_{11}}}\left\{1-\Phi (\frac{{\bar{y}}_{1}(\Delta_{11}+\Delta_{2}{)}^{k}-x_{11}}{\sqrt{\Delta_{2}}}-\theta_{1}\sqrt{\Delta_{2}})\right\}\phi (\frac{x_{11}-\Delta_{11}\theta_{1}}{\sqrt{\Delta_{11}}})dx_{11}}{\sqrt{\Delta_{11}}\left\{1-\Phi (l_{1}-\theta_{1}\sqrt{\Delta_{11}})\right\}}. \end{array}$$

For situations where either $S^{*}=\{1\}$ or $S^{*}=\{1,2\}$ a confidence interval for $\theta_{1}$ conditional on $1\in S^{*}$ can be constructed in a similar manner, except conditioning on $X_{12}=x_{12}$ then has an impact. Specifically, if $x_{12}\leq l_{1}\sqrt{\Delta_{12}}$ , implying $S^{*}=\{1\}$ , then $\Omega_{1}^{u}({\bar{y}}_{1}\Delta_{11}^{k};x_{12})$ is as above and $p_{1}$ and $p_{2}$ stay the same. However, if $x_{12}>l_{1}\sqrt{\Delta_{12}}$ , then the decision to stop at stage 1 is based on $X_{10}$ , and hence $\Omega_{1}^{u}({\bar{y}}_{1}\Delta_{11}^{k};x_{12})=\{x_{1}:x_{1}>(u_{1}\sqrt{\Delta_{11}+\Delta_{12}}-x_{12})\vee {\bar{y}}_{1}\Delta_{11}^{k}\},$ and $\Omega_{1}^{o2}=\{x_{1}:l_{1}\sqrt{\Delta_{11}}<x_{1}\leq u_{1}\sqrt{\Delta_{11}+\Delta_{12}}-x_{12}\}$ provided $u_{1}\sqrt{\Delta_{11}+\Delta_{12}}-x_{12}>l_{1}\sqrt{\Delta_{11}}$ , and is empty otherwise. Let ${\tilde{u}}_{1}=\left(\frac{u1\sqrt{\Delta_{11}+\Delta_{12}}-x_{12}}{\sqrt{\Delta_{11}}}\vee l_{1}\right)$ then the resulting expressions for $p_{1}$ and $p_{2}$ will be the same as above, except we replace $u_{1}$ with ${\tilde{u}}_{1}$ .

*Unconditional p -values for $\theta_{1}$
*

Using the same definition of ${\tilde{u}}_{1}$ as above, for the unconditional p -value function for $\theta_{1}$ ,

$$\Omega_{1}^{\nu 1}({\bar{y}}_{1}\Delta_{11}^{k};x_{12})=\left\{ \begin{array}{ll} \left({\bar{y}}_{1}\Delta_{11}^{k},\infty \right) & \text{if}\;{\bar{y}}_{1}\Delta_{11}^{k}\geq {\tilde{u}}_{1}\sqrt{\Delta_{11}}\\ \left({\tilde{u}}_{1}\sqrt{\Delta_{11}},\infty \right) & \text{if}\;l_{1}\Delta_{11}^{1/2}\leq {\bar{y}}_{1}\Delta_{11}^{k}<{\tilde{u}}_{1}\Delta_{11}^{1/2}\\ \left({\bar{y}}_{1}\Delta_{11}^{k},l_{1}\sqrt{\Delta_{11}}]\cup ({\tilde{u}}_{1}\sqrt{\Delta_{11}},\infty \right) & \text{otherwise.} \end{array}\right.$$

Hence

$$\begin{array}{rl} p_{1}({\bar{y}}_{1};\theta_{1}) & =I(l_{1}>{\bar{y}}_{1}\Delta_{11}^{k-0.5})\times \left\{\Phi (l_{1}-\theta_{1}\sqrt{\Delta_{11}})-\Phi ({\bar{y}}_{1}\Delta_{11}^{k-0.5}-\theta_{1}\sqrt{\Delta_{11}})\right\}\\ & \;+\Phi (\theta_{1}\sqrt{\Delta_{11}}-({\tilde{u}}_{1}\vee {\bar{y}}_{1}\Delta_{11}^{k-0.5})). \end{array}$$

Similarly, $\Omega_{1}^{v2}(x_{12})=(l_{1}\sqrt{\Delta_{11}},{\tilde{u}}_{1}\sqrt{\Delta_{11}})$ and so

$$\begin{array}{rl} & p_{2}({\bar{y}}_{1};\theta_{1})=\\ & \int_{l_{1}\sqrt{\Delta_{11}}}^{{\tilde{u}}_{1}\sqrt{\Delta_{11}}}\left\{1-\Phi (\frac{{\bar{y}}_{1}(\Delta_{11}+\Delta_{20}{)}^{k}-x_{11}}{\sqrt{\Delta_{20}}}-\theta_{1}\sqrt{\Delta_{20}})\right\}\phi (\frac{x_{11}-\Delta_{11}\theta_{1}}{\sqrt{\Delta_{11}}})/\sqrt{\Delta_{11}}dx_{11}, \end{array}$$

which is identical to the numerator in the conditional case.

Analogous expressions for a confidence interval for $\theta_{2}$ , unconditionally or conditional on $S^{*}=\{2\}$ will have the same form except using ${\bar{y}}_{2},\theta_{2},\Delta_{12}$ and $x_{11}$ in place of ${\bar{y}}_{1},\theta_{1},\Delta_{11}$ and $x_{12}$ .

*
P -value function for $\theta_{0}$
*

For the p -value function for $\theta_{0}$ conditional on $S^{*}=\{1,2\}$ , let $\Delta_{1}=\Delta_{11}+\Delta_{12}$ and let $f_{1|0}(x;\theta_{0})$ represent the distribution of $X_{1}=X_{11}+X_{12}$ conditional on $S^{*}=\{1,2\}$ . Here

$$f_{1|0}(x;\theta_{0})=\frac{\int_{l_{1}\sqrt{\Delta_{11}}}^{x-l_{2}\sqrt{\Delta_{12}}}\phi (\frac{x-\Delta_{11}\theta_{0}}{\sqrt{\Delta_{11}}})\phi (\frac{x_{12}-\Delta_{12}\theta_{0}}{\sqrt{\Delta_{12}}})/\sqrt{\Delta_{11}\Delta_{12}}dx_{12}}{\left\{1-\Phi (l_{1}-\theta \sqrt{\Delta_{11}})\}\{1-\Phi (l_{1}-\theta \sqrt{\Delta_{12}})\right\}}$$

for $x\geq l_{1}(\sqrt{\Delta_{11}}+\sqrt{\Delta_{12}})$ and is 0 otherwise. Then

(A.2)
$$p_{1}({\bar{y}}_{0};S^{*}=\{1,2\},\theta_{0})=\int_{l^{*}}^{\infty }f_{1|0}(x|S,\theta_{0})dx,$$

where $l^{*}={\bar{y}}_{0}(\Delta_{11}+\Delta_{12}{)}^{k}\vee u_{1}\sqrt{\Delta_{11}+\Delta_{12}}$ .

Moreover, since the stage 2 information in the design is fixed given $S^{*}=\{1,2\}$ ,

(A.3)
$$p_{2}({\bar{y}}_{S};S^{*}=\{1,2\},\theta_{0})=\int_{l_{1}(\sqrt{\Delta_{11}}+\sqrt{\Delta_{12}})}^{u_{1}(\sqrt{\Delta_{11}+\Delta_{12}})}f_{1|0}(x_{1}|S,\theta_{0})\{1-\Phi (\frac{{\bar{y}}_{S}(\Delta_{1}+\Delta_{20}{)}^{k}-\theta \Delta_{20}-x_{1}}{\sqrt{\Delta_{20}}})\}dx_{1}.$$
