# Supplementary material for: Confidence intervals and point estimates for treatment effects in adaptive enrichment designs
Source: Stat Methods Med Res. 2026 Feb 23;35(4):827–46. doi: 10.1177/09622802261423180 (PMC13161503; doi:10.1177/09622802261423180)
Supplement: sj-pdf-1-smm-10.1177_09622802261423180 - Supplemental material for Confidence intervals and point estimates for treatment effects in adaptive enrichment designs [file sj-pdf-1-smm-10.1177_09622802261423180.pdf]

Supplementary Material: Confidence intervals and point estimates for treatment effects in adaptive enrichment designs

Journal Title
XX(X):1–14
©The Author(s) 2024
Reprints and permission:
sagepub.co.uk/journalsPermissions.nav
DOI: 10.1177/ToBeAssigned
www.sagepub.com/
SAGE

S1 Time-to-event endpoint

As a further simulation scenario, we consider an adaptive enrichment trial with a time-to-event endpoint. The treatment is assumed to act on the hazards such that the hazard experienced by patients in subgroup group j on the treatment arm, T, are related to those in the control group C via a proportional hazards relationship: $h_{Tj}(t) = h_{Cj}(t)\exp(-\theta_j)$ where potentially each subgroup group has a separate hazard function. Hence θ_j corresponds to the log-hazard ratio for subgroup j comparing control to treatment, where the parametrization is chosen to imply $\theta > 0$ corresponds to a treatment benefit. Under this formulation, interest lies in testing $H_0 : \theta_j \leq 0$ versus $H_1 : \theta_j > 0$ for $j = 1, 2$ where the score statistics are the subgroup specific log-rank statistics for the test equality of hazards between treatment groups. Similarly, the Fisher information is taken as the estimated variance of the unstandardized log-rank statistic.

For comparability with the scenarios for normally-distributed responses, we again assume subgroup prevalences of 60% and 40%, respectively and that the aim is to design a trial with 90% power to reject at least one hypothesis in the case where $\boldsymbol{\theta} = (0.2, 0.2)$. Hence the decision boundaries, (l_1, u_1, u_2), for the Magnusson-Turnbull design are the same as before. Similarly, under an assumption that the baseline hazards in each subgroup are similar, the Fisher information for θ_1 and θ_2 after r total events have been observed is approximately $I_1 = 0.6r/4$ and $I_2 = 0.4r/4$[1]. Hence there is a sample size requirement of 625 per stage, but this refers to the number of observed events rather than patients. The first interim analysis is triggered once the 625th event is observed, and if applicable, the second interim analysis occurs at the 1250th event. Note that it is assumed that the rate

at which patients of each subgroup can be recruited cannot change, meaning the second interim will tend to occur later if only one group is enriched at the second stage.

In the simulations, patients are recruited as a Poisson process with overall rate of 1000 per year (600 and 400 per year for subgroups 1 and 2, respectively). The time-to-event distributions under the control are exponential with rates of $\exp(-0.1)$ and $\exp(-0.2)$ in the subgroups 1 and 2, and so under the treatment they are exponential with rates $\exp(-0.1 - \theta_1)$ and $\exp(-0.2 - \theta_2)$.

We consider score ordering and also approximate MLE ordering where we take $\tilde{\theta}_j = Y_j / I_j$ which is a valid asymptotic approximation of the MLE for 'small' θ_j [2].

The unconditional confidence intervals and estimators are assessed on the basis of 10000 simulated repetitions of the trial for each scenario. For the conditional intervals and estimators, as in the main paper, rejection sampling is used to obtain 10000 repetitions in which $\mathcal{S} = \{1\}, \mathcal{S} = \{2\}$ and $\mathcal{S} = \{1, 2\}$, respectively, for each of the scenarios.

It should be noted that the above scenario results in a very large trial. To give an indication of the performance of the methods for a more realistically sized survival trial, a second scenario is considered that corresponds to a trial with 80% power to reject at least one hypothesis in the case where $\boldsymbol{\theta} = (0.3, 0.3)$, but where otherwise the above assumptions are the same. In this case the target sample size is 210 events per stage. Patients are again recruited as a Poisson process, but with an overall rate of 360 per year, in order to give an similar trial timescale.

S1.1 Results

Tables S1, S2 and S3 give the coverage of conditional one-sided confidence intervals for the selected group when subgroup 1, subgroup 2, and both subgroups are chosen for the trial aiming for 90% power. Generally, both score-ordered and MLE-ordered confidence intervals have satisfactory coverage. There is some indication of further-from-nominal coverage for the cases where subgroup 2 is selected. This may be due to the observed information in each period being different from $I_1 = 0.6r/4$ and $I_2 = 0.4r/4$ due to different baseline hazards for each subgroup. Specifically, a greater proportion of the first 625 events occur in subgroup 1 due to the higher hazard and hence $I_1 > 0.6 \times 625/4$ and $I_2 < 0.4 \times 625/4$. However, if both groups are enriched in the second stage, a greater proportion of subgroup 2 patients from stage 1 are left to carry-over to stage 2 and hence the ratio of I_1 to I_2 is less pronounced in the second stage. An alternative set of scenarios (not presented) where the baseline hazard is assumed the same in subgroups 1 and 2 gave coverage probabilities closer to nominal.

Mirroring the results for normally distributed responses, the score-ordered intervals tend to have slightly better power to exclude 0 than MLE-ordering. Naive confidence intervals based on the MLE given by a Cox proportional hazards model (stratified by subgroup for $\hat{\theta}_S$) and corresponding Fisher information tend to under-cover, with the under coverage being substantial in null scenarios.

Note that in the case where $\theta = (0.2, 0)$, the population-level treatment effect, θ_S, is not well-defined because the proportional hazards model is not subgroup mixable [3]. The

Table S1. Coverage and power (Type I error for null case) of conditional one-sided 97.5% confidence intervals when subgroup 1 is selected, under different scenarios for θ for Magnusson-Turnbull design with a time-to-event endpoint designed to obtain 90% to detect a log-hazard ratio of 0.2.

	Coverage probability			Power		
Scenario	score	mle	naive	score	mle	naive
$\theta = (0, 0)$	0.9770	0.9771	0.9211	0.0230	0.0229	0.0789
$\theta = (0.2, 0)$	0.9762	0.9756	0.9685	0.7075	0.6983	0.9075
$\theta = (0.2, 0.2)$	0.9777	0.9751	0.9706	0.7499	0.7384	0.9243

Table S2. Coverage and power (Type I error for null case) of conditional one-sided 97.5% confidence intervals when subgroup 2 is selected, under different scenarios for θ for Magnusson-Turnbull design with a time-to-event endpoint designed to obtain 90% to detect a log-hazard ratio of 0.2.

	Coverage probability			Power		
Scenario	score	mle	naive	score	mle	naive
$\theta = (0, 0)$	0.9761	0.9760	0.9223	0.0239	0.0240	0.0777
$\theta = (0.2, 0)$	0.9774	0.9784	0.9258	0.0226	0.0216	0.0742
$\theta = (0.2, 0.2)$	0.9763	0.9758	0.9613	0.6701	0.6434	0.8815

Table S3. Coverage and power (Type I error for null case) of conditional one-sided 97.5% confidence intervals when both subgroups are selected, under different scenarios for θ for Magnusson-Turnbull design with a time-to-event endpoint designed to obtain 90% to detect a log-hazard ratio of 0.2.

	Coverage probability			Power		
Scenario	score	mle	naive	score	mle	naive
$\theta = (0, 0)$	0.9740	0.9735	0.8145	0.0260	0.0265	0.1855
$\theta = (0.2, 0)$	0.9714	0.9634	0.9200	0.3432	0.2535	0.7997
$\theta = (0.2, 0.2)$	0.9754	0.9762	0.9705	0.6615	0.5202	0.9772

treatment effect could be quantified through some form of average hazard ratio[4,5]. For instance, using the concordance odds definition, $\theta_S = 0.1194$. For simplicity, coverage, bias and RMSE in the tables are just calculated on the basis of the prevalence-weighted average 0.12.

Tables S4-S6 give the corresponding results for the smaller trial size of 210 events per stage. Generally the coverage probabilities continue to be close to the nominal 97.5% and there is no discernible deterioration in performance due to the smaller total number of events.

Tables S7, S8 and S9 give the mean bias, median bias and root-mean squared error for the median unbiased estimate (MUE), conditional moment estimate (CME) and naive MLE for the selected group. The MUE and CME are constructed using MLE-ordered

Table S4. Coverage and power (Type I error for null case) of conditional one-sided 97.5% confidence intervals when subgroup 1 is selected, under different scenarios for θ for Magnusson-Turnbull design with a time-to-event endpoint designed to obtain 80% to detect a log-hazard ratio of 0.3.

	Coverage probability			Power		
Scenario	score	mle	naive	score	mle	naive
$\theta = (0, 0)$	0.9749	0.9744	0.9180	0.0251	0.0256	0.0820
$\theta = (0.3, 0)$	0.9748	0.9735	0.9628	0.5521	0.5276	0.8061
$\theta = (0.3, 0.3)$	0.9730	0.9746	0.9667	0.6215	0.5975	0.8560

Table S5. Coverage and power (Type I error for null case) of conditional one-sided 97.5% confidence intervals when subgroup 2 is selected, under different scenarios for θ for Magnusson-Turnbull design with a time-to-event endpoint designed to obtain 80% to detect a log-hazard ratio of 0.3.

	Coverage probability			Power		
Scenario	score	mle	naive	score	mle	naive
$\theta = (0, 0)$	0.9737	0.9748	0.9263	0.0252	0.0263	0.0737
$\theta = (0.3, 0)$	0.9713	0.9718	0.9193	0.0282	0.0287	0.0807
$\theta = (0.3, 0.3)$	0.9732	0.9738	0.9553	0.5047	0.5352	0.7948

Table S6. Coverage and power (Type I error for null case) of conditional one-sided 97.5% confidence intervals when both subgroups are selected, under different scenarios for θ for Magnusson-Turnbull design with a time-to-event endpoint designed to obtain 80% to detect a log-hazard ratio of 0.3.

	Coverage probability			Power		
Scenario	score	mle	naive	score	mle	naive
$\theta = (0, 0)$	0.9732	0.9731	0.8201	0.0268	0.0269	0.1799
$\theta = (0.3, 0)$	0.9770	0.9725	0.9229	0.2417	0.1674	0.7037
$\theta = (0.3, 0.3)$	0.9756	0.9766	0.7037	0.5585	0.3921	0.9414

p-value functions. The MUE and CME generally perform comparably with similar root mean squared errors. The MUE tends to be close to median-unbiased and has a small mean-bias whereas the reverse pattern holds for the CME. The MLE is consistently positively biased, with the greatest bias occurring when groups are selected under the null scenario. Since the MLE tends to have a lower variance than either the MUE or CME, the RMSE of MLE is often comparable to the others, but is lower when both groups are correctly chosen and notably higher when both groups are chosen under the null. The bias of the population treatment effect in the case where $\theta = (0.2, 0)$ and $\mathcal{S} = \{1, 2\}$ is lower than in the normal case. This may be because the combined data from the survival trials have a greater proportion of information from group 2 due to the higher number of

expected events, which causes the degree of positive bias (seen in the normal case) to be attenuated back towards 0.

Table S7. Performance of point estimators for θ_1 when subgroup 1 is selected for Magnusson-Turnbull design with a time-to-event endpoint designed to obtain 90% to detect a log-hazard ratio of 0.2. MUE=Median unbiased estimate, CME=Conditional moment estimate, MLE=naive maximum likelihood estimate. MUE and CME are computed based on p-value functions using MLE ordering ($k = 1$).

	Mean bias			Median bias			RMSE		
Scenario	MUE	CME	MLE	MUE	CME	MLE	MUE	CME	MLE
$\theta = (0, 0)$	0.0015	-0.0038	0.0475	0.0007	-0.0039	0.0443	0.0796	0.0769	0.0778
$\theta = (0.2, 0)$	0.0119	0.0019	0.0315	0.0021	-0.0094	0.0203	0.0896	0.0898	0.0910
$\theta = (0.2, 0.2)$	0.0115	0.0021	0.0298	0.0014	-0.0081	0.0204	0.0860	0.0863	0.0871

Table S8. Performance of point estimators for θ_2 when subgroup 2 is selected for Magnusson-Turnbull design with a time-to-event endpoint designed to obtain 90% to detect a log-hazard ratio of 0.2. MUE=Median unbiased estimate, CME=Conditional moment estimator, MLE=naive maximum likelihood estimate. MUE and CME are computed based on p-value functions using MLE ordering ($k = 1$).

	Mean bias			Median bias			RMSE		
Scenario	MUE	CME	MLE	MUE	CME	MLE	MUE	CME	MLE
$\theta = (0, 0)$	0.0025	-0.0037	0.0468	0.0000	-0.0056	0.0423	0.0886	0.0853	0.0882
$\theta = (0.2, 0)$	0.0001	-0.0049	0.0431	-0.0013	-0.0064	0.0394	0.0817	0.0788	0.0809
$\theta = (0.2, 0.2)$	0.0151	0.0054	0.0370	0.0051	-0.0175	0.0177	0.1006	0.0995	0.1052

Table S9. Performance of point estimators for θ_S when both groups are selected for Magnusson-Turnbull design with a time-to-event endpoint designed to obtain 90% to detect a log-hazard ratio of 0.2. MUE=Median unbiased estimate, CME=Conditional moment estimator, MLE=naive maximum likelihood estimate. MUE and CME are computed based on p-value functions using MLE ordering ($k = 1$). Assumed true value of $\theta_S = 0.12$ used when $\theta = (0.2, 0)$.

	Mean bias			Median bias			RMSE		
Scenario	MUE	CME	MLE	MUE	CME	MLE	MUE	CME	MLE
$\theta = (0, 0)$	0.0049	-0.0031	0.0725	0.0015	-0.0075	0.0660	0.0818	0.0800	0.0929
$\theta = (0.2, 0)$	0.0185	0.0107	0.0549	0.0106	0.0117	0.0467	0.0860	0.0868	0.0885
$\theta = (0.2, 0.2)$	0.0058	0.0041	0.0316	-0.0014	-0.0033	0.0289	0.0759	0.0760	0.0700

Table S10. Coverage and power of unconditional simultaneous confidence intervals for $\theta = (\theta_1, \theta_2)$ for Magnusson-Turnbull design with a time-to-event endpoint designed to obtain 90% to detect a log-hazard ratio of 0.2. Power refers to the proportion of intervals that exclude 0 for at least one component.

	Coverage probability		Power	
Scenario	score	MLE	score	MLE
$\theta = (0, 0)$	0.9785	0.9786	0.0215	0.0214
$\theta = (0.2, 0)$	0.9775	0.9745	0.6943	0.6870
$\theta = (0.2, 0.2)$	0.9759	0.9767	0.7641	0.7419

Table S11. Coverage and power of unconditional simultaneous confidence intervals for $\theta = (\theta_1, \theta_2)$ for Magnusson-Turnbull design with a time-to-event endpoint designed to obtain 80% to detect a log-hazard ratio of 0.3. Power refers to the proportion of intervals that exclude 0 for at least one component.

	Coverage probability		Power	
Scenario	score	MLE	score	MLE
$\theta = (0, 0)$	0.9760	0.9745	0.0240	0.0255
$\theta = (0.3, 0)$	0.9737	0.9752	0.5428	0.5403
$\theta = (0.3, 0.3)$	0.9738	0.9750	0.6672	0.6418

Table S12. Coverage and power of conditional simultaneous confidence intervals for $\theta = (\theta_1, \theta_2)$ when $\mathcal{S}^* = \{1, 2\}$ for Magnusson-Turnbull design with a time-to-event endpoint designed to obtain 90% to detect a log-hazard ratio of 0.2. Power refers to the proportion of intervals that exclude 0 for at least one component.

	Coverage probability		Power	
Scenario	score	MLE	score	MLE
$\theta = (0, 0)$	0.9764	0.9805	0.0236	0.0195
$\theta = (0.2, 0)$	0.9740	0.9757	0.4234	0.2721
$\theta = (0.2, 0.2)$	0.9753	0.9763	0.5032	0.3817

Tables S10 and S11 give the coverage of unconditional simultaneous 97.5% confidence intervals for the two sample sizes of trial. In all cases the coverage is reasonably close to nominal. Again, score-ordered intervals have slightly higher power.

Tables S12 and S13 give the coverage of conditional simultaneous 97.5% confidence intervals in the cases where both subgroups are selected. As above, there is no apparent deterioration of performance for a smaller sample size, but there is a tendency for the MLE-based intervals to be conservative and to have considerably less power than the score based intervals.

Table S13. Coverage and power of conditional simultaneous confidence intervals for $\theta = (\theta_1, \theta_2)$ when $\mathcal{S}^* = \{1, 2\}$ for Magnusson-Turnbull design with a time-to-event endpoint designed to obtain 80% to detect a log-hazard ratio of 0.3. Power refers to the proportion of intervals that exclude 0 for at least one component.

	Coverage probability		Power	
Scenario	score	MLE	score	MLE
$\theta = (0, 0)$	0.9753	0.9800	0.0247	0.0200
$\theta = (0.2, 0)$	0.9739	0.9746	0.2972	0.1765
$\theta = (0.2, 0.2)$	0.9749	0.9766	0.4027	0.2794

S2 Lin *et al* design

Lin *et al*[6] proposed adaptive enrichment designs with a sample size re-estimation element. Specifically, they consider similar stage 1 stopping rules for efficacy and futility as Magnusson-Turnbull. However, if either or both groups are enriched at stage 2, the stage 2 sample size and critical value are chosen in order to simultaneously fix the conditional Type I error based on a conditional error function which increases depending on the value of the stage 1 statistic, and to fix the estimated conditional power (substituting the estimated treatment effect based on the stage 1 data) to some constant value. As a result, the stage 2 sample size depends on the specific values of X_{11} and X_{12}, rather than just the region Ω_j in which $\mathbf{X}_1$ lies. The design has the advantage of leading to low stage 2 sample sizes in cases where the stage 1 statistic is close to the stage 1 efficacy stopping boundary, u_1. However, this is at the cost of potentially requiring a very large stage 2 sample size when the stage 1 statistic is only just above the stage 1 futility stopping boundary, l_1.

Lin *et al* based their decisions on the Wald statistics, but these are asymptotically equivalent to the score statistic (and identical in the case of normally distributed responses). In terms of decisions at the end of stage 1, the Lin *et al* design only differs in the decision made in the case where both groups' statistics exceed l_1 but only one group's exceeds u_1. For the Magnusson-Turnbull design this always leads to $\mathcal{S}^* = \{1, 2\}$, whereas in Lin *et al* if the combined statistic is below u_1, the trial will stop for efficacy with $\mathcal{S}^*$ corresponding only to the group with the individual statistic above u_1. Figure S1 depicts the resulting regions Ω_j, where j ranges from 1 to 7. In these regions, region 1 signifies the absence of subgroup selection, region 2 denotes the enrichment of only subgroup 1, region 3 signifies the enrichment of only subgroup 2, region 4 indicates the simultaneous enrichment of both subgroups, region 5 signifies the presence of subgroup 1 for efficacy, region 6 signifies the presence of subgroup 2 for efficacy, and region 7 indicates the presence of both subgroups for efficacy in stage 1. The control of the type I error rate is achieved through the employment of the circular error function proposed by Proschan and Hunsberger[7]. This function, denoted as $A(x_1, l_1, u_1)$,

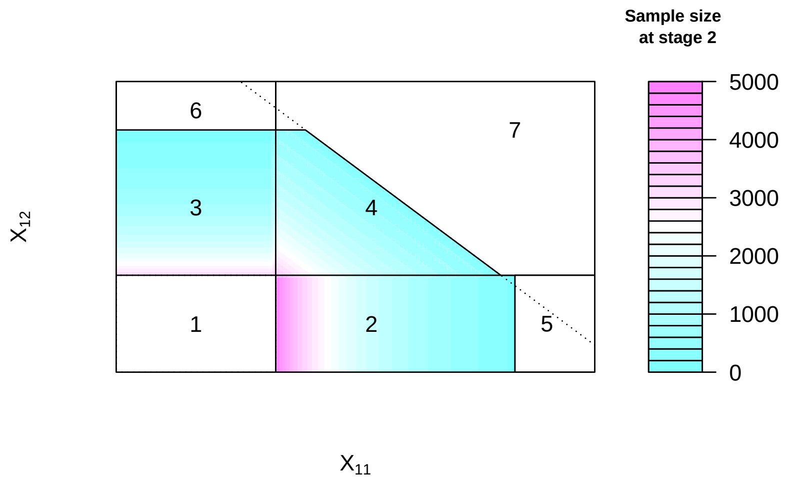

Figure S1. Example of the region of the stage 1 standardized score statistics. The contoured area represents sample spaces of the first stage statistics in which the trial proceeds to the second stage.

is defined as follows:

$$A(x_1, l_1, u_1) = \begin{cases} 0, & \text{if } x_1 \leq l_1. \\ 1 - \Phi(\sqrt{u_1^2 - x_1^2}), & \text{if } l_1 < x_1 < u_1. \\ 1, & \text{if } x_1 \geq l_1. \end{cases}$$

Here, x_1 represents the first stage statistic.

The property that Lin et al. design will always stop at stage 1 provided at least one group's standardized statistic exceeds u_1 means that $\Omega_1^o(x_{12})$ and $\Omega_1^u(\bar{y}_1\sqrt{\Delta_{11}}; x_{12})$ are not invariant to the value of x_{12}, and $\mathcal{S}^* = \{1\}$ will not necessarily occur for the highest values of x_{11}. Therefore, more appropriate intervals are achieved by calculating conditional on $j \in \mathcal{S}^*$ rather than $\mathcal{S}^* = \{1\}$. Since the stage 2 sample size can in some cases be substantially larger than stage 1, using score ordering $(k = 0.5)$ leads to non-monotonic p-value functions with respect to θ, whereas those based on MLE ordering $(k = 1)$ are well-behaved.

Assuming interest is in a confidence interval for θ_1, we can note that the values of x_{11} for which group 1 is selected depend heavily on x_{12}. For instance, if $x_{12} > u_1\sqrt{\Delta_{12}}$ then group 1 is only selected if the combined standardized score statistic exceeds u_1. Let

$$\tilde{l}_1 = \begin{cases} l_1\sqrt{\Delta_{11}} \vee (u_1\sqrt{\Delta_{11} + \Delta_{12}} - x_{12}) & \text{if } x_{12} \geq u_1\sqrt{\Delta_{12}} \\ l_1\sqrt{\Delta_{11}} & \text{otherwise,} \end{cases}$$

then $\Omega_1^s(x_{12}) = (\tilde{l}_1, \infty)$. Similarly, let $l_2^* = u_1(\sqrt{\Delta_{11}+\Delta_{12}} - \sqrt{\Delta_{11}}) \vee l_1\sqrt{\Delta_{12}}$ and

$$\tilde{u}_1 = \begin{cases} u_1\sqrt{\Delta_{11}} & \text{if } x_{12} < l_2^* \\ l_1\sqrt{\Delta_{11}} \vee (u_1\sqrt{\Delta_{11}+\Delta_{12}} - x_{12}) & \text{otherwise,} \end{cases}$$

then $\Omega_1^u(\bar{y}_1\Delta_{11}^k, x_{12}) = (\tilde{u}_1 \vee \bar{y}_1\Delta_{11}^k, \infty)$ and hence $p_1(\bar{y}_1; 1 \in \mathcal{S}^*, \theta_1, x_{12}) = M_1/M_0$ where

$$M_1 = \Phi(\sqrt{\Delta_{11}}\theta_1 - (\tilde{u}_1 \vee \bar{y}_1\Delta_{11}^k)/\sqrt{\Delta_{11}})$$
$$M_0 = \Phi(\sqrt{\Delta_{11}}\theta_1 - \tilde{l}_1/\sqrt{\Delta_{11}}).$$

The trial can only proceed to stage 2 with group 1 if $x_{12} < u_1\sqrt{\Delta_{12}}$. Hence we have that $p_2(\bar{y}_1; 1 \in \mathcal{S}^*, \theta_1, x_{12}) = M_2/M_0$ for $x_{12} < u_1\sqrt{\Delta_{12}}$ where

$$M_2 = \int_{\tilde{l}_1}^{\tilde{u}_1} \Phi\left(\theta_1\sqrt{\Delta_{21}(\mathbf{x}_1)} - \{\bar{y}_1(\Delta_{11}+\Delta_{21}(\mathbf{x}_1))^k - x_{11}\}/\sqrt{\Delta_{21}(\mathbf{x}_1)}\right) f_{11}(x_{11})dx_{11}$$

for $x_{12} < u_1\sqrt{\Delta_{12}}$ and where the form of $\Delta_{21}(\mathbf{x}_1)$ for $\mathbf{x}_1 = (x_{11}, x_{12})$ depends on whether $x_{12} > l_1\sqrt{\Delta_{12}}$ (i.e. whether both groups or only group 1 are enriched).

For an unconditional p-value function for group 1, we can note that group 1 will be dropped from the design at stage 1 if $x_{11} < \tilde{l}_1$ (as defined above). Hence

$$p_1(\bar{y}_1; \theta_1, x_{12}) = M_1 + I(\bar{y}_1\sqrt{\Delta_{11}} < \tilde{l}_1)M_3$$

where $M_3 = \Phi(\tilde{l}_1/\sqrt{\Delta_{11}} - \theta_1\sqrt{\Delta_{11}}) - \Phi(\bar{y}_1\Delta_{11}^{k-0.5} - \theta_1\sqrt{\Delta_{11}})$ and $p_2(\bar{y}_1; \theta_1, x_{12}) = M_2$ as defined above.

When $\mathcal{S}^* = \{1, 2\}$, a confidence interval for θ_0, assuming $\boldsymbol{\theta} = (\theta_0, \theta_0)$, can be constructed using a similar construction to Magnusson and Turnbull's design given in the Appendix of the main paper. The only differences are that Δ_2 in the equation for p_2 has dependence on $\mathbf{x}_1$ and that the form of $f_{1|0}(x \mid \theta_0)$ is more complicated.

Hence we define

$$T_j = \int_{l_1\sqrt{\Delta_{1j}}}^{u_1(\sqrt{\Delta_{10}}-\sqrt{\Delta_{1j'}})} f_{1j}(x_{1j})\left\{\Phi(\tilde{u}_1(x_{1j})/\sqrt{\Delta_{1j'}} - \theta_0\Delta_{1j'}) - \Phi(u_1 - \theta_0\Delta_{1j'})\right\} dx_{1j},$$

for $j = 1, 2$, where $\tilde{u}_1(x_{1j}) = u_1\sqrt{\Delta_{1j'}} \vee (u_1\sqrt{\Delta_1} - x_{1j})$ and $\Delta_{10} = \Delta_{11} + \Delta_{12}$, and

$$T_{12} = \Phi(\theta_0\sqrt{\Delta_{11}} - l_1)\Phi(\theta_0\sqrt{\Delta_{12}} - l_1).$$

Then $M_4 = P(\mathcal{S}^* = \{1, 2\} \mid \theta_0) = T_{12} - T_1 - T_2$, gives the denominator for the conditional density.

Table S14. Coverage and power (Type I error for null case) of conditional one-sided 97.5% confidence intervals for Lin et al's design when subgroup 1 is selected, under different scenarios for θ.

	Coverage probability		Power	
Scenario	MLE	naive	MLE	naive
$\theta = (0, 0)$	0.9751	0.8683	0.0249	0.1317
$\theta = (0.2, 0)$	0.9763	0.9609	0.2926	0.9929
$\theta = (0.2, 0.2)$	0.9773	0.9617	0.2886	0.9936

Next, let

$$\tilde{l}_0(y) = \begin{cases} l_1\sqrt{\Delta_{11}} & \text{if } y < l_1\sqrt{\Delta_{11}} + u_1\sqrt{\Delta_{12}} \\ y - u_1\sqrt{\Delta_{12}} & \text{if } l_1\sqrt{\Delta_{11}} + u_1\sqrt{\Delta_{12}} \le y < u_1\sqrt{\Delta_{10}} \\ l_1\sqrt{\Delta_{11}} & \text{if } y \ge u_1\sqrt{\Delta_{10}} \end{cases}$$

and

$$\tilde{u}_0(y) = \begin{cases} u_1\sqrt{\Delta_{11}} \wedge (y - l_1\sqrt{\Delta_{12}}) & \text{if } y < u_1\sqrt{\Delta_{10}} \\ y - l_1\sqrt{\Delta_{12}} & \text{if } y \ge u_1\sqrt{\Delta_{10}}, \end{cases}$$

then $f_{1|0}(y \mid \theta_0) = \{\int_{\tilde{l}_0(y)}^{\tilde{u}_0(y)} f_{11}(x) f_{12}(y - x)dx\}/M_4$. With this definition of $f_{1|0}(y \mid \theta_0)$, $p_1(\bar{y}_0; \theta_0; \mathcal{S}^* = \{1, 2\})$ and $p_2(\bar{y}_0; \theta_0; \mathcal{S}^* = \{1, 2\})$ are as given in equations (A.2) and (A.3) of the main paper, respectively.

S2.1 Simulation setups

The parameters for Lin *et al*'s design in the simulations are chosen to minimize the sum of the expected sample size under the null and expected sample size under $\theta = (0.2, 0.2)$, subject to a constraint that the power to reject any null when $\theta = (0.2, 0.2)$ exceeds 90%. For comparability with the Magnusson-Turnbull design, the stage 1 sample size is fixed at 625 and the prevalence of subgroup 1 is 0.6. As suggested by Lin *et al*, the design is found using simulation. This results in parameters $l_1 = 1.15, u_1 = 2.5056, \beta_2 = 0.089$.

S2.2 Results

Tables S14, S15, S16 give the coverage and power of conditional one-sided 97.5% confidence intervals for Lin *et al*'s design using MLE ordering for the p-value functions. In each case, the 97.5% confidence interval based on inversion of the MLE ordering p-value function is compared with a naive Wald interval directly using the MLE and Fisher information. In general, the coverage of the intervals based on MLE ordering have close to nominal coverage. The only exception is the interval for θ_S when $\theta = (0.2, 0)$, where the assumption that $\theta_1 = \theta_2$ is not met. In general, the MLE ordered intervals have substantially lower power than the corresponding Magnusson-Turnbull intervals. This is primarily due to the higher value of l_1 used in the design.

Table S15. Coverage and power (Type I error for null case) of conditional one-sided 97.5% confidence intervals for Lin et al's design when subgroup 2 is selected, under different scenarios for θ.

Scenario	Coverage probability		Power	
	MLE	naive	MLE	naive
$\theta = (0, 0)$	0.9721	0.8766	0.0279	0.1234
$\theta = (0.2, 0)$	0.9731	0.8817	0.0269	0.1183
$\theta = (0.2, 0.2)$	0.9746	0.9404	0.1959	0.9877

Table S16. Coverage and power (Type I error for null case) of conditional one-sided 97.5% confidence intervals for Lin et al's design when both subgroups are selected, under different scenarios for θ.

Scenario	Coverage probability		Power	
	MLE	naive	MLE	naive
$\theta = (0, 0)$	0.9752	0.5467	0.0248	0.4533
$\theta = (0.2, 0)$	0.9533	0.8190	0.2202	0.9553
$\theta = (0.2, 0.2)$	0.9733	0.9479	0.3742	0.9966

Tables S17, S18 and S19 give the mean bias, median bias and root-mean squared error for the median unbiased estimate (MUE), conditional moment estimate (CME) and naive MLE for the selected group. The results have a broadly similar pattern to those for the Magnusson-Turnbull design. However, the MLE tends to have a greater bias for Lin et al's design meaning the MUE and CME have a lower RMSE in all cases except for the combined estimate when $\theta = (0.2, 0.2)$. For the CME for the combined treatment effect in Table S19, due to the computation required, the CME values are based on an approximation invovling computing the expected combined MLE on a fine grid of values of θ_S assuming Δ_{11} and Δ_{12} are fixed at their expected values, rather than using the observed values of Δ_{11} and Δ_{12} in each trial.

Table S17. Performance of point estimators for θ_1 for Lin et al's design when subgroup 1 is selected. MUE=Median unbiased estimate, CME=Conditional moment estimate, MLE=naive maximum likelihood estimate. MUE and CME are computed based on p-value functions using MLE ordering ($k = 1$).

	Mean bias			Median bias			RMSE		
Scenario	MUE	CME	MLE	MUE	CME	MLE	MUE	CME	MLE
$\theta = (0, 0)$	0.015	0.000	0.039	0.000	-0.015	0.023	0.072	0.070	0.081
$\theta = (0.2, 0)$	0.021	0.005	0.046	0.000	-0.014	0.033	0.080	0.080	0.086
$\theta = (0.2, 0.2)$	0.019	0.003	0.045	-0.003	-0.017	0.030	0.079	0.080	0.085

Table S18. Performance of point estimators for θ_2 for Lin et al's design when subgroup 2 is selected. MUE=Median unbiased estimate, CME=Conditional moment estimate, MLE=naive maximum likelihood estimate. MUE and CME are computed based on p-value functions using MLE ordering ($k = 1$).

	Mean bias			Median bias			RMSE		
Scenario	MUE	CME	MLE	MUE	CME	MLE	MUE	CME	MLE
$\theta = (0, 0)$	0.020	0.002	0.041	0.000	-0.018	0.021	0.087	0.083	0.096
$\theta = (0.2, 0)$	0.019	0.001	0.041	0.000	-0.018	0.021	0.085	0.082	0.094
$\theta = (0.2, 0.2)$	0.031	0.006	0.056	0.000	-0.028	0.026	0.098	0.097	0.109

Table S19. Performance of point estimators for θ_S for Lin et al's design when both groups are selected. MUE=Median unbiased estimate, CME=Conditional moment estimator, MLE=naive maximum likelihood estimate. MUE and CME are computed based on p-value functions using MLE ordering ($k = 1$). Assumed true value of $\theta_S = 0.12$ used when $\theta = (0.2, 0)$.

	Mean bias			Median bias			RMSE		
Scenario	MUE	CME	MLE	MUE	CME	MLE	MUE	CME	MLE
$\theta = (0, 0)$	0.024	0.008	0.114	0.000	-0.026	0.092	0.100	0.109	0.141
$\theta = (0.2, 0)$	0.031	0.027	0.097	0.028	0.030	0.103	0.100	0.099	0.118
$\theta = (0.2, 0.2)$	0.001	-0.001	0.053	0.000	-0.009	0.048	0.090	0.085	0.079

Table S20. Coverage and power of conditional and unconditional simultaneous and confidence intervals for $\theta = (\theta_1, \theta_2)$ for Lin et al's design. Power refers to the proportion of intervals that exclude 0 for at least one component.

	conditional		unconditional	
Scenario	coverage	power	coverage	power
$\theta = (0, 0)$	0.9722	0.0278	0.9743	0.0257
$\theta = (0.2, 0)$	0.9764	0.7397	0.9726	0.2202
$\theta = (0.2, 0.2)$	0.9722	0.7887	0.9728	0.3061

Table S20 gives the coverage and power of simultaneous unconditional confidence intervals and simultaneous confidence intervals conditional on $\mathcal{S}^* = \{1, 2\}$. In both cases, the coverages are close to nominal. For the conditional confidence intervals, there is again lower power than the corresponding Magnusson-Turnbull intervals.

References

1. Di Scala L and Glimm E. Time-to-event analysis with treatment arm selection at interim. *Statistics in Medicine* 2011; 30(26): 3067–3081.
2. Jennison C and Turnbull BW. *Group sequential methods with applications to clinical trials*. CRC Press, 1999.
3. Ding Y, Lin HM and Hsu JC. Subgroup mixable inference on treatment efficacy in mixture populations, with an application to time-to-event outcomes. *Statistics in Medicine* 2016; 35(10): 1580–1594.
4. Kalbfleisch JD and Prentice RL. Estimation of the average hazard ratio. *Biometrika* 1981; 68(1): 105–112.
5. Rauch G, Brannath W, Brückner M et al. The average hazard ratio–a good effect measure for time-to-event endpoints when the proportional hazard assumption is violated? *Methods of Information in Medicine* 2018; 57(03): 089–100.
6. Lin R, Yang Z, Yuan Y et al. Sample size re-estimation in adaptive enrichment design. *Contemporary Clinical Trials* 2021; 100: 106216.
7. Proschan MA and Hunsberger SA. Designed extension of studies based on conditional power. *Biometrics* 1995; 51(4): 1315–1324.
