# Supplementary material for: Confidence intervals and point estimates for treatment effects in adaptive enrichment designs
Source: Stat Methods Med Res. 2026 Feb 23;35(4):827–46. doi: 10.1177/09622802261423180 (PMC13161503; doi:10.1177/09622802261423180)
Supplement: sj-pdf-2-smm-10.1177_09622802261423180 - Supplemental material for Confidence intervals and point estimates for treatment effects in adaptive enrichment designs [file sj-pdf-2-smm-10.1177_09622802261423180.pdf]

Confidence intervals for adaptive enrichment designs: R routines

Jinyu Zhu, Andrew Titman, Fang Wan

2025-09-12

The following document presents R functions for calculating confidence intervals and corrected point estimates using the examples in the main paper as data examples.

Magnusson-Turnbull design

Function to calculate the p-value associated with a selected group (conditional on selection). See the first section of the Appendix of the main paper.

```
mt_pval_cond <- function(x, ybar, l1, u1, D11, D2, k=1) {
  #x: Value of \theta for the selected group
  #ybar: Observed (standardized) test statistic for the selected group
  #l1: Futility boundary at stage 1 (on the standardized score scale)
  #u1: Superiority boundary at stage 1 (on the standardized score scale)
  #D11: Observed information for selected group at stage 1.
  #D2: Expected information for selected group if the design proceeded to stage 2
  #k: Ordering parameter used for the p-value/computation of ybar.
  m <- max(u1, ybar*D11^k/D11^0.5)
  #Put on standardized score scale
  p1 <- exp(pnorm(x*sqrt(D11) - m, log=TRUE) - pnorm(x*sqrt(D11) - l1, log=TRUE))
  if (u1>l1) {
    p2 <- (integrate(function(y) pnorm(x*sqrt(D2) + y/sqrt(D2)
           - ybar*(D11+D2)^k/sqrt(D2))*dnorm((y - D11*x)/sqrt(D11)),
         l1*sqrt(D11), u1*sqrt(D11))$value/sqrt(D11))/pnorm(x*sqrt(D11) - l1)
  }else{
    p2 <- 0
  }
  p1 + p2
}
```

Function to calculate the p-value associated with an individual group (unconditional on selection). See the second second of the Appendix of the main paper.

```
mt_pval_uncond <- function(x, ybar, l1, u1, D11, D2, k=1) {
  m <- max(u1, ybar*D11^k/D11^0.5)
  p1 <- pnorm(x*sqrt(D11) - m) + (l1 > ybar*D11^k/D11^0.5)*(pnorm(l1 - x*sqrt(D11)) -
                                                  pnorm(ybar*D11^k/D11^0.5 - x*sqrt(D11)))
  p2 <- (integrate(function(y) pnorm(x*sqrt(D2) + y/sqrt(D2) -
            ybar*(D11+D2)^k/sqrt(D2))*dnorm((y - D11*x)/sqrt(D11)), l1*sqrt(D11),
                   u1*sqrt(D11))$value/sqrt(D11))
  p1 + p2
}
```

Function to find lower or upper intervals via p-value function inversion

```r
find_interval <- function(q, ybar, l1, u1, D11, D2, type="cond",range=c(-3,3), k=1) {
  #q: Desired quantile, e.g. 0.025 for a 1-sided 97.5% confidence interval
  if (type=="cond") {
    if (mt_pval_cond(range[1],ybar,l1,u1,D11,D2,k) > q) return(range[1])
    root <- uniroot(function(x) mt_pval_cond(x, ybar, l1,u1,D11,D2,k) - q,
                       interval=range)$root
  }
  if (type=="uncond") {
    if (mt_pval_uncond(range[1],ybar,l1,u1,D11,D2) > q) return(range[1])
    root <- uniroot(function(x) mt_pval_uncond(x, ybar, l1,u1,D11,D2,k) - q,
                      interval=range)$root
  }
  return(root)
}
```

Panitumumab-FOLFIRI trial example

We first input the observed stagewise log-rank test statistics.

```r
X11 <- 13.04
X12 <- -0.87
D11 <- 22.80
D12 <- 26.29
X21 <- 9.94
D21 <- 51.26
```

We also need the decision boundary limits at stage 1.

```r
l1 = 0.519
u1 = 2.748
```

Confidence interval for wild-type KRAS group, conditional on selection

In the panitumumab-FOLFIRI example, group 1 (Wild-type KRAS) is selected and proceeds to stage 2. The $\bar{y}$ value for group 1 is then given by:

```r
X1 <- X11 + X21
ybar <- X1/(D11+D21)
```

where here MLE ordering is chosen. The lower and upper limits of the 95% confidence interval for the log-hazard ratio of wild-type KRAS tumour patients, conditional on selection is then:

```r
lower <- find_interval(0.025, ybar, l1, u1, D11, D21, k=1)
upper <- find_interval(0.975, ybar, l1, u1, D11, D21, k=1)
c(lower, upper)
```

```
## [1] 0.01483726 0.52585634
```

Note here, since the log-rank test statistics are orientated such that a positive X_{ij} corresponds to a treatment benefit, the bounds correspond to negative log-hazard ratios (i.e. the log-hazard ratio of control compared to experimental treatment). Hence here the 95% confidence interval is (-0.526, -0.015).

Unconditional simultaneous confidence intervals

To construct simultaneous confidence intervals for the treatment effects in the Wild-type and not Wild-type groups we find separate 97.5% confidence intervals for each group using the unconditional method. For the unconditional method, the superiority boundaries may need to be adjusted. In our case, the boundary for

group 1 does not need to be adjusted because group 2's statistic did not exceed l_1. However, group 2's statistic does not adjusting to give the value of $X_{12}/\sqrt{D_{12}}$ required for the pooled statistic to exceed $u1$

```
#Intervals for group 1
lower1 <- find_interval(0.0125, ybar, l1, u1, D11, D21, type="uncond",k=1)
upper1 <- find_interval(0.9875, ybar, l1, u1, D11, D21, type="uncond",k=1)

#Adjusted upper boundary for group 2:
u1tilde <- max((u1*sqrt(D11+D12) - X11)/sqrt(D12),l1)
#MLE statistic for group 2
ybar2 <- X12/D12

lower2 <- find_interval(0.0125, ybar2, l1, u1tilde, D12, D12, type="uncond",k=1)
upper2 <- find_interval(0.9875, ybar2, l1, u1tilde, D12, D12, type="uncond",k=1)
```

This results in $(-0.609, -0.036) \times (-0.404, 0.461)$ as given in the main paper.

Computation of MUE and CME

To calculate the median unbiased estimate (MUE), it is only necessary to specify $q = 0.5$ within the find_interval function. Hence the MUE for wild type KRAS conditional on selection is given by

```
mue1 <- find_interval(0.5, ybar, l1, u1, D11, D21, type="cond",k=1)
mue1
```

```
## [1] 0.2837824
```

where, as above, this corresponds to a log-HR estimate of -0.284.

The following functions enable calculation of the conditional moment estimator as detailed in Section 2.7 of the main paper.

```
solve_CME <- function(ybar, l1, u1, D11, D2, k=1, discr=0.2, approx=NULL) {
  #ybar: observed statistic, e.g. MLE or standardized score
  #l1, u1: stage 1 limits
  #D11: stage 1 Fisher information
  #D2: Expected stage 2 Fisher information
  #k: ordering parameter
  #discr: Size of interval about est/approx in which to search for the solution.
  #approx: Optional approximate solution to use as a starting point.
  #      : for k \neq 1, should set this to be a good estimate of \theta e.g. the MLE
  if (is.null(approx)) approx <- ybar
  uniroot(function(x) expec_cond(x,l1,u1,D11,D2,k) - ybar,
             c(approx - discr, min(approx+discr, ybar+0.01)))$root
}

expec_cond <- function(x, l1,u1,D11,D2,k=0.5,ymax=5) {
  #Function to compute the two integrals
  #ymax: Value at which to truncate the integrals
  low <-integrate(function(u) sapply(u,function(v) mt_pval_cond(x, v, l1,u1,D11,D2,k)),
                  0,ymax)$value
  up <-integrate(function(u) sapply(u,function(v) 1 - mt_pval_cond(x, v, l1,u1,D11,D2,k)),
                  -ymax,0)$value
  low - up
}
```

To calculate the CME for the Wild-type KRAS subgroup, we can then use:

```r
cme <- solve_CME(ybar, l1, u1, D11, D21, k=1, discr=0.2)
cme
```

```
## [1] 0.260093
```

which corresponds to a log-HR of -0.260.

Confidence intervals for combined groups

In Section 2.4.3 and the third section of the Appendix of the main paper, details of the p-value function for the overall effect θ_0, conditional on $\mathcal{S}^* = \{1,2\}$ is given. Computation in this case is more involved because it requires integration with respect to $f_{1|0}(x;\theta_0)$, the distribution of the combined stage 1 score statistic conditional on selection of the whole group at stage 1, which is itself not available in closed form.

The following functions facilitate computation of the conditional p-value functions.

```r
#Function to calculate the density of the combined score statistic
# conditional on selection
f1S <- function(v,x,l1,D11,D12) {
  #v: combined statistic value
  #x: assumed common theta value
  #l1: selection bound
  #D11, D12: stage 1 Fisher infomation for groups 1 and 2.
  if (v <= l1*(sqrt(D11) + sqrt(D12))) return(0)
  lcond <- pnorm(l1*sqrt(D11),x*D11, sqrt(D11),lower.tail =FALSE ,log.p=TRUE) +
             pnorm(l1*sqrt(D12),x*D12, sqrt(D12),lower.tail =FALSE ,log.p=TRUE)
  maxv <- v - l1*sqrt(D12)
  p<-safeintegrate(function(z) dnorm(z, x*D11, sqrt(D11))*dnorm(v - z, x*D12,
                                              sqrt(D12)),l1*sqrt(D11), maxv)$value
  exp(log(p) - lcond)
}

#Combined p-value function when both groups chosen
mt_pval_cond2 <- function(x, ybar, l1, u1, D11, D12, D2, k=1) {
  condp <- pnorm(x*sqrt(D11) - l1)*pnorm(x*sqrt(D12) - l1)
  minv <- l1*(sqrt(D11) + sqrt(D12))
  maxv <- max(ybar *(D11 + D12)^k,u1*sqrt(D11 + D12))
  if (maxv > minv) {
    p1 <- 1 - safeintegrate(function(z) sapply(z, function(v) f1S(v,x,l1,D11,D12)),
                              minv,maxv)$value
    p2 <- safeintegrate(function(z) sapply(z, function(v)
            f1S(v,x,l1,D11,D12)*(1 - pnorm(ybar*(D11 + D12 + D2)^k - v, D2*x,
                sqrt(D2)))), minv, u1*sqrt(D11 + D12))$value
  }else{
    p1 <- 1
    p2 <-0
  }
  p1 + p2
}

#Function to increase integral subdivisions, if necessary.
safeintegrate <- function(f, lower, upper, ..., subdivisions=100 ,
                          rel.tol=.Machine$double.eps^0.25, abs.tol=rel.tol) {
  computed <- FALSE
  nit <- 0
  while (!computed) {
```

```r
    nit <- nit+1
    try1 <- tryCatch(integrate(f, lower, upper, ...,subdivisions=subdivisions,
                rel.tol=rel.tol,abs.tol=abs.tol), error =function(e) return("error"))
    if (identical(try1,"error")) {
      subdivisions <- subdivisions+50
      rel.tol <- rel.tol*1.5
      abs.tol <- rel.tol
    }else{
      computed <- TRUE
      #if (nit>1) print(paste("Needed",nit,"iterations to integrate"))
      return(try1)
    }
    if (nit >100) stop("Unable to get sufficient subdivisions.")
  }
}

#Function to invert the combined group p-value to obtain lower or upper bounds
# on confidence intervals
find_interval2 <- function(q, ybar, l1, u1, D11, D12, D2, range=c(-3,3), k=1) {
  #q: Quantile of interest e.g. 0.025 for a 1-sided 97.5% CI
  #ybar, l1, u1: as above
  #D11, D12: stage 1 observed information for groups 1 and 2
  #D2: Expected combined stage 2 information.
  #range: Range of values in which to seek a root for the interval bound
  #k: ordering parameter.
  if (mt_pval_cond2(range[1],ybar,l1,u1,D11,D12,D2,k) > q) return(range[1])
  root <- uniroot(function(x) mt_pval_cond2(x, ybar, l1, u1, D11,D12,D2,k) -q,
                    interval=range)$root
  return(root)
}
```

In the panitumumab-FOLFIRI trial, only the wild-type subgroup was selected. However, we can consider a hypothetical situation where instead the not Wild type also gave promising results at stage 1, e.g. $X_{12} = 10.0$ rather than -0.87. For simplicity, we suppose the stage 1 information and the group 1 statistic is unchanged. In that $X_{12}/\sqrt{D_{12}} > l_1$ and $X_{11}/\sqrt{D_{11}} > l_1$, so $\mathcal{S}^* = \{1, 2\}$ and moreover, $(X_{11} + X_{12})/\sqrt{D_{11} + D_{12}} = 3.289 > u_1$. As a consequence, the trial would stop for efficacy at the end of stage 1.

```r
X12a = 10 #New value for group 2's stage 1 statistic
ybar <- (X11 + X12a)/(D11+D12)
lower12 <- find_interval2(0.025, ybar,l1,u1,D11,D12,D11+D12, k=1)
upper12 <- find_interval2(0.975, ybar,l1,u1,D11,D12,D11+D12, k=1)
c(lower12,upper12)
```

```
## [1] 0.1180976 0.7320258
```

Point estimate for combined groups

The methods for computing the MUE and CME for the combined group effect are directly analogous.

For the MUE, we can directly use find_interval2:

```r
mue12 <- find_interval2(0.5, ybar,l1,u1,D11,D12,D11+D12, k=1)
mue12
```

```
## [1] 0.4408646
```

Finding the CME is relatively computationally intensive since it requires performing a line search over a

function which itself involves triple numerical integration.

```
solve_CME2 <- function(ybar, l1, u1, D11, D12, D2, k=1, discr=0.2, approx=NULL) {
  if (is.null(approx)) approx <- ybar
  uniroot(function(x) expec_cond2(x,l1,u1,D11,D12,D2,k) - ybar, c(approx - discr,
                                           min(approx+discr, ybar+0.01)))$root
}

expec_cond2 <- function(x, l1,u1,D11,D12,D2,k=0.5,maxy=5) {
  low <- integrate(function(u) sapply(u,function(v)
              mt_pval_cond2(x, v, l1,u1,D11,D12,D2,k)),0,maxy)$value
  up <- integrate(function(u) sapply(u,function(v)
            1 - mt_pval_cond2(x, v, l1,u1,D11,D12,D2,k)),-maxy,0)$value
  low - up
}

cme12 <- solve_CME2(ybar,l1,u1,D11,D12,D11+D12,k=1)
cme12
```

```
## [1] 0.4262748
```

Lin et al design

In Section S2 of the Supplementary Material, the adaptive enrichment design of Lin et al (2021) is used as a second example. In this section, *R* functions for computing the intervals are provided and illustrated using a simulated example.

```
#Circular error function
A_cir <- function(t, l, u) {
  (1 - pnorm(sqrt(pmax(0,u^2 - t^2))))*((l < t) & (t <u)) + 1*(t >= u)
}


#Function to compute the stage 2 sample size given the stage 1 statistic
computeN2_0 <- function(tg, l,u,N1, b2,tol=1e-8) {
  tg[(tg==l)]<-tg[(tg==l)] + tol
  tg[(tg==u)]<-tg[(tg==u)] - tol
  A<-A_cir(tg,l,u)
  N1 *((qnorm(1-A)+qnorm(1-b2))/tg)^2
}


#Function to calculate the p-value for a given group conditional on selection.
#Here it is conditional on j \in S
lin_pval_cond <- function(x, ybar, l1, u1, D11, D12, x12, b2, k=1) {
  #NB: x12 is unstandardized score at stage 1
  #Limit for the conditional probability of selection given x12
  llim <- l1 *(x12 < u1*sqrt(D12)) +
          max(l1, (u1*sqrt(D11 + D12) - x12)/sqrt(D11))*(x12 >= u1*sqrt(D12))
  lstar <- max(u1*(sqrt(D11+D12) - sqrt(D11)), l1*sqrt(D12))
  ulim1 <- u1*(x12 < lstar) + llim*(x12 >= u1*sqrt(D12)) +
  max(l1,min(u1, (u1*sqrt(D11 + D12) - x12)/sqrt(D11)))*(x12 < u1*sqrt(D12) & x12>=lstar)
  m <- max(ulim1, ybar*D11^k/D11^0.5)
  p1 <- exp(pnorm(x*sqrt(D11) - m, log=TRUE) - pnorm(x*sqrt(D11) - llim, log=TRUE))
  if (ulim1 > llim) {
```

```
    if (x12 < l1*sqrt(D12)) {
      p2 <- (integrate(function(y) { D2 <- computeN2_0(y/sqrt(D11), l1, u1,
          (D11+D12), b2) ; pnorm(x*sqrt(D2) + y/sqrt(D2) -
        ybar*(D11+D2)^k/sqrt(D2))*dnorm((y - D11*x)/sqrt(D11))}, llim*sqrt(D11),
          ulim1*sqrt(D11))$value/sqrt(D11))/pnorm(x*sqrt(D11) - llim)
    }else{
      p2 <- (integrate(function(y) {D2 <- computeN2_0((x12 + y)/sqrt(D11 + D12), l1, u1,
        (D11+D12), b2) * (D11/(D11+D12)) ; pnorm(x*sqrt(D2) + y/sqrt(D2) -
        ybar*(D11+D2)^k/sqrt(D2))*dnorm((y - D11*x)/sqrt(D11))}, llim*sqrt(D11),
          ulim1*sqrt(D11))$value/sqrt(D11))/pnorm(x*sqrt(D11) - llim)
    }
  }else{
    p2<-0
  }
  p1 + p2
}

lin_pval_uncond <- function(x, ybar, l1, u1, D11, D12, x12, b2, k=1) {
  if (x12 < u1*sqrt(D12)) {
    #Range where stop for efficacy at stage 1.
    llim <- l1*sqrt(D11)
    #Need to consider the case where llim > ulim - this implies that the value
    # of X12 ensures will always go to stage 2
    ulim <- max(llim, (x12 < l1*sqrt(D12))*(u1*sqrt(D11)) +
            (x12>=l1*sqrt(D12))*min(u1*sqrt(D11+D12) - x12, u1*sqrt(D11)))
    p1 <- (ybar*D11^k < llim)*(pnorm(llim, x*D11, sqrt(D11)) -
    pnorm(ybar*D11^k, x*D11,sqrt(D11))) +
            (1 - pnorm(max(ybar*D11^k, ulim),x*D11,sqrt(D11)))
    #p2 involves an integral over the range llim to ulim
    if (ulim > llim) {
      if (x12 >= l1*sqrt(D12)) {
        p2 <- integrate(function(y) { D2 <- computeN2_0((x12 + y)/sqrt(D11 + D12),
             l1, u1, (D11+D12), b2) * (D11/(D11+D12)) ; pnorm(x*sqrt(D2) + y/sqrt(D2) -
          ybar*(D11+D2)^k/sqrt(D2))*dnorm((y - D11*x)/sqrt(D11))},
              llim, ulim)$value/sqrt(D11)
      }else{
  p2 <- integrate(function(y) { D2 <- computeN2_0(y/sqrt(D11), l1, u1, (D11+D12), b2) ;
  pnorm(x*sqrt(D2) + y/sqrt(D2) - ybar*(D11+D2)^k/sqrt(D2))*dnorm((y - D11*x)/sqrt(D11))},
          llim, ulim)$value/sqrt(D11)
      }
    }else{
      p2 <- 0
    }
  }else{
    p1 <- (1- pnorm(ybar*D11^k, x*D11, sqrt(D11)))
    p2 <- 0
  }
  return(p1+p2)
}

#Function to find the lower or upper limit of confidence intervals
find_interval_lin <- function(q, ybar, l1, u1, D11, D12, X12, b2, type="cond",
                              range=c(-3,3), k=1) {
```

```
  if (type=="cond") {
    if (lin_pval_cond(range[1],ybar,l1,u1,D11,D12, X12, b2 ,k) > q) return(range[1])
    root <- uniroot(function(x) lin_pval_cond(x, ybar, l1,u1,D11,D12, X12, b2, k) - q,
                    interval=range)$root
  }
  if (type=="uncond") {
    if (lin_pval_uncond(range[1],ybar,l1,u1,D11,D12, X12, b2 ,k) > q) return(range[1])
    root <- uniroot(function(x) lin_pval_uncond(x, ybar, l1,u1,D11,D12, X12, b2, k) - q,
                    interval=range)$root
  }
  return(root)
}
```

Example: Group 1 selected at stage 1

Suppose, the same design as in Section S2.1. is followed, where the stage 1 sample size is fixed at 625, the prevalence of subgroup 1 is 0.6, the stage 1 thresholds are $l_1 = 1.15$ and $u_1 = 2.5056$ and β_2, which sets the conditional power, is set a $\beta_2 = 0.089$.

First, suppose in the trial the stage 1 statistics are $X_{11} = 20.48$ and $X_{12} =4.51 $ with corresponding information $\Delta_{11} = 94.2$ and $\Delta_{12} = 62.1$. This leads to selection of only group 1 for stage 2.

```
X11=20.48 ; X12=4.51; D11=94.2; D12=62.1
N1=625; l=1.15; u=2.5056;b2=0.089
tg = X11/sqrt(D11)
  A<-A_cir(tg,l,u)
  N2<-N1 *((qnorm(1-A)+qnorm(1-b2))/tg)^2
  c_alp <- (tg^2 + qnorm(1-A)*(qnorm(1-A) + qnorm(1-b2)))/sqrt(tg^2 +
                                           (qnorm(1-A) + qnorm(1-b2))^2)
c(N2,c_alp)
```

```
## [1] 1021.805675    2.364209
```

Based on the desire, to retain conditional power, the second stage sample size is set at 1022 and the stage 2 threshold is set at 2.364. Suppose in the second stage, the test statistic is $X_{21} = 43.3$ and $\Delta_{21} = 253$. Based on MLE ordering, this gives an overall statistic of $\bar{y} = (20.48 + 43.3)/(94.2 + 253) = 0.184$, with a corresponding Wald statistic of $3.42 > 2.364$, meaning we conclude efficacy for group 1.

We are firstly interested in obtaining a confidence interval for the selected group.

```
X21=43.3; D21=253
ybar = (X11+X21)/(D11+D21)
lower1 <- find_interval_lin(0.025, ybar, l, u, D11, D12, X12, b2, k=1)
upper1 <- find_interval_lin(0.975, ybar, l, u, D11, D12, X12, b2, k=1)
c(lower1,upper1)
```

```
## [1] -0.06010244  0.26026271
```

Hence the conditional interval does not exclude zero and hence disagrees with the trial result. However, this is in line with findings of the simulation study where the power of the CIs was quite low.

In addition, we are also interested in the simultaneous unconditional confidence interval for the two treatment effects.

```
lower1u <- find_interval_lin(0.0125, ybar, l, u, D11, D12, X12, b2, type="uncond",k=1)
upper1u <- find_interval_lin(0.9875, ybar, l, u, D11, D12, X12, b2, type="uncond",k=1)
ybar2 <- X12/D12
lower2u <- find_interval_lin(0.0125, ybar2, l, u, D12, D11, X11, b2, type="uncond",k=1)
```

```
upper2u <- find_interval_lin(0.9875, ybar2, l, u, D12, D11, X11, b2, type="uncond",k=1)
c(lower1u,upper1u,lower2u,upper2u)
```

```
## [1]  0.02259914  0.35151001 -0.20768195  0.35747008
```

In this case, the simultaneous 95% confidence interval does exclude 0 for θ_1 and hence agrees with the overall trial result.

Point estimation for a single group

A corresponding MUE for θ_1 can be obtained using find_interval_lin

```
mue_lin <- find_interval_lin(0.5, ybar, l, u, D11, D12, X12, b2, k=1)
mue_lin
```

```
## [1] 0.1590503
```

Similarly, a CME can be obtained via integration of the p-value function in the same way as the Magnusson-Turnbull case.

```
solve_CME_lin <- function(ybar, l1, u1, D11, D12, x12, b2, k=0.5,
                          discr=0.2, approx=NULL) {
  if (is.null(approx)) approx <- ybar
  uniroot(function(x) expec_cond_lin(x,l1,u1,D11,D12,x12,b2, k) - ybar,
                    c(approx - discr, min(approx+discr, ybar+0.01)))$root
}

expec_cond_lin <- function(x, l1,u1,D11,D12,x12,b2,k=0.5, maxy=5) {
  low <- safeintegrate(function(u) sapply(u,function(v)
                lin_pval_cond(x, v, l1,u1,D11,D12,x12,b2,k)),0,maxy)$value
  up <- safeintegrate(function(u) sapply(u,function(v)
              1 - lin_pval_cond(x, v, l1,u1,D11,D12,x12,b2,k)),-maxy,0)$value
  low - up
}

cme_lin <- solve_CME_lin(ybar, l, u, D11, D12, X12, b2, k=1)
cme_lin
```

```
## [1] 0.1375591
```

Example: Both groups selected

Now suppose the same Lin et al design was run, but the stage 1 statistics were instead $X_{11} = 14.48$ and $X_{12} = 9.51$ with corresponding information $\Delta_{11} = 94.2$ and $\Delta_{12} = 62.1$. This leads to selection of only group 1 for stage 2. In this case, both groups have individual Wald statistics between l_1 and u_1. The combined Wald statistic is $(14.48 + 9.51)/\sqrt{(94.2 + 62.1)} = 1.92 < u_1 = 2.5056$ so both groups proceed to stage 2.

```
X11=14.48 ; X12=9.51; D11=94.2; D12=62.1
N1=625; l=1.15; u=2.5056;b2=0.089
tg = (X11+X12)/sqrt(D11+D12)
  A<-A_cir(tg,l,u)
  N2<-N1 *((qnorm(1-A)+qnorm(1-b2))/tg)^2
  c_alp <- (tg^2 + qnorm(1-A)*(qnorm(1-A) + qnorm(1-b2)))/sqrt(tg^2 +
                                              (qnorm(1-A) + qnorm(1-b2))^2)
c(N2,c_alp)
```

```
## [1] 1485.278349    2.395977
```

In this case, the conditional power calculation leads to a second stage sample size of 1486, with a stage 2 threshold of 2.396.

Suppose, at stage 2 the observed statistics are $X_{21} = 31.8$ and $X_{22} = 29.7$ with corresponding information $\Delta_{21} = 223$ and $\Delta_{22} = 148.6$. This leads to an overall Wald statistic of $(X_{11} + X_{12} + X_{21} + X_{22})/(\Delta_{11} + \Delta_{12} + \Delta_{21} + \Delta_{22}) = 3.72 > 2.396$. Hence the trial concludes superiority for both groups.

```
X21=31.8 ; X22=29.7; D21=223; D22=148.6
y = (X11+X12+X21+X22)/sqrt(D11+D12+D21+D22)
```

Firstly, the same approach as before can be used to compute the simultaneous unconditional confidence intervals.

```
ybar1 <- (X11+X21)/(D11+D21)
ybar2 <- (X12+X22)/(D12+D22)
lower1u <- find_interval_lin(0.0125, ybar1, l, u, D11, D12, X12, b2, type="uncond",k=1)
upper1u <- find_interval_lin(0.9875, ybar1, l, u, D11, D12, X12, b2, type="uncond",k=1)
lower2u <- find_interval_lin(0.0125, ybar2, l, u, D12, D11, X11, b2, type="uncond",k=1)
upper2u <- find_interval_lin(0.9875, ybar2, l, u, D12, D11, X11, b2, type="uncond",k=1)
c(lower1u,upper1u,lower2u,upper2u)
```

```
## [1] -0.01522554  0.35078358 -0.02887561  0.43362877
```

In this case, the simultaneous confidence interval contains $(\theta_1, \theta_2) = (0, 0)$.

However, given $\mathcal{S}^* = \{1, 2\}$ interest also lies in obtaining a confidence interval for the combined effect, θ_0. The following functions are used to obtain a conditional confidence interval for the combined effect.

```
#Function for the density of the stage 1 statistic conditional on selection of both groups.
f1S_lin <- function(v,x,l1,u1,D11,D12) {
  if (v < l1*(sqrt(D11) + sqrt(D12))) return(0)
  cond0 <- pnorm(x*sqrt(D11) - l1)*pnorm(x*sqrt(D12) - l1) #Prob both statistics > l1
  #But have to calculate the triangle probabilities.
  tri1 <- integrate(function(z) dnorm(z, x*D11,sqrt(D11))*(pnorm(pmax(u1*sqrt(D12),
       u1*sqrt(D11 +D12) - z), x*D12,sqrt(D12)) - pnorm(u1*sqrt(D12), x*D12,sqrt(D12))),
              l1*sqrt(D11), max(l1*sqrt(D11), u1*(sqrt(D11+D12) - sqrt(D12))))$value
  tri2 <- integrate(function(z) dnorm(z, x*D12,sqrt(D12))*(pnorm(pmax(u1*sqrt(D11),
       u1*sqrt(D11 +D12)- z), x*D11,sqrt(D11)) - pnorm(u1*sqrt(D11), x*D11,sqrt(D11))),
              l1*sqrt(D12), max(l1*sqrt(D12), u1*(sqrt(D11+D12) - sqrt(D11))))$value
  condp <- cond0 - tri1 - tri2  #integrate over values of x1
  lims <- linelims(v,l1,u1,D11,D12)
  p<-integrate(function(z) dnorm(z, x*D11, sqrt(D11))*dnorm(v - z, x*D12,sqrt(D12)),
                  lims[1], lims[2])$value
  p/condp
}

#Auxiliary function to determine the interval along which to integrate
linelims <- function(v,l1,u1,D11,D12) {
  if (v > u1*(sqrt(D11 + D12))) {
    lower <- l1*sqrt(D11)
    upper <- v - l1*sqrt(D12)
    return(c(lower,upper))
  }
  if (v < l1*(sqrt(D11)) + u1*sqrt(D12)) {
    lower <- l1*sqrt(D11)
    upper <- min(u1*sqrt(D11), v-l1*sqrt(D12))
    return(c(lower,upper))
```

```
  }
  if (v>= l1*(sqrt(D11)) + u1*sqrt(D12) & v<= u1*(sqrt(D11 + D12))) {
    lower <- v - u1*sqrt(D12)
    upper <- min(u1*sqrt(D11) , v-l1*sqrt(D12))
    return(c(lower,upper))
  }
}

#Combined group version
lin_pval_cond2 <- function(x, ybar, l1, u1, D11, D12, b2, k=1) {
  minv <- l1*(sqrt(D11) + sqrt(D12))
  maxv <- max(ybar *(D11 + D12)^k,u1*sqrt(D11 + D12))
  p1 <- 1 - integrate(function(z) sapply(z, function(v) f1S_lin(v,x,l1,u1,D11,D12)),
                    minv,maxv)$value
  D2 <- function(y) { computeN2_0(y/sqrt(D11 + D12), l1, u1, (D11+D12), b2) }
  p2 <- integrate(function(z) sapply(z, function(v) f1S_lin(v,x,l1,u1,D11,D12)*(1 -
      pnorm(ybar*(D11 + D12 + D2(v))^k - v, D2(v)*x, sqrt(D2(v)))) ), minv,
              u1*sqrt(D11 + D12))$value
  p1 + p2
}

#Function for finding the interval limits when both groups are selected.
find_interval_lin2 <- function(q, ybar, l1, u1, D11, D12, b2, range=c(-3,3), k=1) {
  if (lin_pval_cond2(range[1],ybar,l1,u1,D11,D12,b2, k) > q) return(range[1])
  root <- uniroot(function(x) lin_pval_cond2(x, ybar, l1, u1, D11,D12,b2, k) -q,
                interval=range)$root
  return(root)
}
```

We can then compute the overall MLE estimate $\bar{y}$ and use it in the find_interval_lin2 function

```
ybar12 <- (X11+X12+X21+X22)/(D11+D12+D21+D22)
lower12 <- find_interval_lin2(0.025,ybar12,l, u, D11, D12, b2, range=c(-2,3),k=1)
upper12 <- find_interval_lin2(0.975,ybar12,l, u, D11, D12, b2, range=c(-2,3),k=1)
c(lower12, upper12)
```

```
## [1] -0.2986259  0.2188285
```

Point estimation for the combined effect

As before, the MUE can be directly obtained using the find_interval_lin2 function

```
mue12 <- find_interval_lin2(0.5,ybar12,l, u, D11, D12, b2, range=c(-2,3),k=1)
mue12
```

```
## [1] 0.06404536
```

The CME for the combined effect in a Lin design follows the same approach as Magnusson-Turnbull, but is particularly slow to compute due to the complexity of the form of the $f_{1|0}$ for this design.

```
solve_CME2 <- function(ybar, l1, u1, D11, D12, b2, k=1, discr=0.2, approx=NULL) {
  if (is.null(approx)) approx <- ybar
  uniroot(function(x) expec_cond2(x,l1,u1,D11,D12, b2,k) - ybar,
             c(approx - discr, min(approx+discr, ybar+0.01)))$root
}

expec_cond2 <- function(x, l1,u1,D11,D12,b2,k=1,maxy=5) {
```

```r
  low <- safeintegrate(function(u) sapply(u,function(v)
             lin_pval_cond2(x, v, l1,u1,D11,D12,b2,k)),0,maxy)$value
  up <- safeintegrate(function(u) sapply(u,function(v)
           1 - lin_pval_cond2(x, v, l1,u1,D11,D12,b2,k)),-maxy,0)$value
  low - up
}

cme12 <- solve_CME2(ybar12, l, u, D11, D12, b2, k=1, approx=mue12)
cme12
```

```
## [1] 0.06187286
```

Here there is close agreement between the CME and MUE estimates, which are both substantially lower than the MLE.
